# Dissecting Community Structure in Wild Blueberry Root and Soil Microbiome

**DOI:** 10.3389/fmicb.2018.01187

**Published:** 2018-06-06

**Authors:** Svetlana N. Yurgel, Gavin M. Douglas, Ashley Dusault, David Percival, Morgan G. I. Langille

**Affiliations:** ^1^Department of Plant, Food, and Environmental Sciences, Dalhousie University, Truro, NS, Canada; ^2^Department of Microbiology and Immunology, Dalhousie University, Halifax, NS, Canada; ^3^Department of Pharmacology, Dalhousie University, Halifax, NS, Canada

**Keywords:** bacterial communities, eukaryotic communities, plant–microbe interaction, *Vaccinium angustifolium*, community networks

## Abstract

A complex network of functions and symbiotic interactions between a eukaryotic host and its microbiome is a the foundation of the ecological unit holobiont. However, little is known about how the non-fungal eukaryotic microorganisms fit in this complex network of host–microbiome interactions. In this study, we employed a unique wild blueberry ecosystem to evaluate plant-associated microbiota, encompassing both eukaryotic and bacterial communities. We found that, while soil microbiome serves as a foundation for root microbiome, plant-influenced species sorting had stronger effect on eukaryotes than on bacteria. Our study identified several fungal and protist taxa, which are correlated with decreased fruit production in wild blueberry agricultural ecosystems. The specific effect of species sorting in root microbiome resulted in an increase in relative abundance of fungi adapted to plant-associated life-style, while the relative abundance of non-fungal eukaryotes was decreased along the soil-endosphere continuum in the root, probably because of low adaptation of these microorganisms to host–plant defense responses. Analysis of community correlation networks indicated that bacterial and eukaryotic interactions became more complex along the soil-endosphere continuum and, in addition to extensive mutualistic interactions, co-exclusion also played an important role in shaping wild blueberry associated microbiome. Our study identified several potential hub taxa with important roles in soil fertility and/or plant–microbe interaction, suggesting the key role of these taxa in the interconnection between soils and plant health and overall microbial community structure. This study also provides a comprehensive view of the role of non-fungal eukaryotes in soil ecosystem.

## Introduction

Plant health and development are fundamentally dependent on the interaction between host and its microbiome. Plants are no longer considered single organisms, but rather dynamic entities comprising both plants and microbiota with complex interactions and functions (Zilber-Rosenberg and Rosenberg, 2008; Vandenkoornhuyse et al., 2015). The recruitment of the plant microbiota suggests a gradual enrichment of soil microbial community (species sorting) through several habitats toward a defined subset of microorganisms occupying internal plant tissue, the endosphere (Vandenkoornhuyse et al., 2015). These habitats are distinguished by physical proximity to the plant and the level of host’s influence on microbial communities. These habitats, from least to most proximal, include bulk soil, the rhizosphere (the thin layer of soil surrounding roots), the rhizoplane (the root tissue surface colonized by microorganisms), and the endosphere.

Soil is the origin of the plant-associated microbiome and, while soil properties are determinants of root microbiome, the host plant significantly influences formation of rhizosphere and endophytic microbial communities. As the understanding of soil and plant microbiome progresses, there is a need to move the focus of the research from a simple description of community structure toward identification of the underlying mechanisms that define complexity of microbiota and link all members of soil community into single, stable, and functional entity (Fierer, 2017; Shade, 2017). As a result, the microbe–microbe and microbe–host interactions in plant microbiomes and their role in shaping root-associated microbial communities, as well as the potential of these interactions to affect host health have been the subject of several recent studies (Agler et al., 2016; Lareen et al., 2016; van Overbeek and Saikkonen, 2016; Busby et al., 2017; Yang et al., 2017; Zhang et al., 2017). The role of some microbial taxa (“hub” taxa) in shaping plant microbiomes in response to abiotic and host-derived factors has been recently proposed and verified (Agler et al., 2016). The hub taxa were defined as microbial groups, which are significantly more connected within the network than other groups based on centrality measurements, such as degree, betweenness centrality and closeness centrality. The strong plant–microbe interactions allow hub taxa to exert indirect effects on microbial communities by affecting the host and triggering plant species sorting preferences. In addition, hub taxa also directly inhibit or facilitate the growth of other microbes affecting overall interconnected communities (Agler et al., 2016).

Bacterial and fungal communities play an important role in soil and plant microbiomes (van Overbeek and Saikkonen, 2016) and in plant nutrient acquisition and stress adaptation (Vandenkoornhuyse et al., 2015). In addition to archaea, bacteria, and fungi, protists are highly abundant in soils and are represented by phylogenetically diverse group. Soil protists carry out a broad range of functions that affect soil fertility and plant health. These functions include modulating bacterial, fungal, algal, and nematode populations (Geisen, 2016; Geisen et al., 2015a), influencing soil nutrient cycling (Seppey et al., 2017), and exerting animal and plant-pathogenic effects (Geisen et al., 2015b). Protists play a central role in linking bacterial and fungal soil populations into a single and complex ecological network (Xiong et al., 2017) with prevalence of antagonistic interactions detected between the kingdoms and mutualistic interactions detected within the kingdoms (Agler et al., 2016). Protistan community structures are consistent within habitat types and geographic regions (Grossmann et al., 2016) and influenced by biotic and abiotic factors (Xiong et al., 2017; Yurgel et al., 2017). Despite their importance in soil ecosystems, protists remain the least characterized microorganisms in soil. In particular, plant-associated protistan communities and their roles in plant–microbiome interaction are not well understood.

*Vaccinium angustifolium* (wild blueberry) management is designed to bring agricultural field standards to natural wild blueberry habitats but retain these conditions close to natural (Hall et al., 1979; Eaton, 1988; Bell et al., 2009; Drummond et al., 2009). We recently used this system to study bacterial, fungal, and non-fungal eukaryotic communities and their responses to biotic and abiotic factors in a separate article. We found that soil bacteria and eukaryotes responded differently to biotic and abiotic factors: soil eukaryotes were more influenced by soil chemical characteristics, while bacteria more strongly responded to the presence of the plant (Yurgel et al., 2017). In this study, we employed wild blueberry managed and natural ecosystems to study interactions between plant and its microbiome focusing on root-associated bacterial and eukaryotic communities, which was mostly comprised by endophytic microorganisms. We evaluated how aggregate differences between forest and managed systems affect root microbiome and identified several eukaryotic taxa linked to field fruit production yield. We also analyzed the inter-kingdom correlations in soil and root-associated microbiota to identify the most connected (hub) microbial taxa in soil and root-associated communities. As a result, this study provides an integrative view on inter-kingdom soil and plant associated communities and identifies microbial taxa with potential importance in plant health and production.

## Materials and Methods

### Sample Collection

The wild blueberry root samples, rhizosphere and bulk soil used in this study were collected in August 2015 (Yurgel et al., 2017). The sample collection, preparation, sequencing and initial bioinformatics analysis of the microbial communities from the rhizosphere and bulk soil was published previously (Yurgel et al., 2017). We included this sequencing data obtained from rhizosphere and bulk soil samples in this study as well for further analysis and comparison with the sequencing data obtained from root samples. Samples used in this study were collected across six managed blueberry fields and two forest sites adjacent to Nova Scotia Blueberry Institute (NSBI) fields at Debert and a commercial field situated near Collingwood Corner, Canada (Yurgel et al., 2017) (Supplementary Table S1). Three managed fields had a history of low yield fruit production and the other three had a history of high fruit yield production. After collecting rhizosphere soils from wild blueberry roots, MngRhizo and FrstRhizo samples (Yurgel et al., 2017), MngRoot and FrstRoot samples were stored in 50 ml sterile Falcon tubes at -86°C. A total of 15 roots from natural (FrstRoot) and 34 roots for manages (MngRoot) plants were collected (Supplementary Table S1).

### Root Tissue Sample Preparation

For DNA isolation around 1 g of each root was placed into a 15 ml tube with 10 ml sterile water and sonicated for 60 s at 20°C in ultrasonic water-bath with 35 kHz frequency. Each root sample was then removed from the tube and placed into a new tube with 10 ml sterile water and sonicated for 60 s again. This step was repeated twice. The cleaned roots were cut into 5 mm pieces using sterile scissors and then placed into sterilized mortals. The samples were frozen in liquid nitrogen and ground into a fine powder using a sterile pestle. A total of 0.250 g ground root tissue was used for DNA isolation.

### DNA Extraction and Sequencing

DNA extraction was carried out using the PowerSoil DNA Isolation Kit (MO BIO Laboratories, Carlsbad, CA, United States) according to the manufacturer’s protocol. The same DNA isolation kit was previously used for DNA extraction from rhizosphere and bulk soil (Yurgel et al., 2017). DNA quality and concentration were measured using a NanoDrop 1000 spectrophotometer (Thermo Scientific, Waltham, MA, United States). At least 50 ng (10 μL) of DNA sample were sent to the Dalhousie University CGEB-IMR^1^ for V6–V8 16S rRNA gene (16S; forward: ACGCGHNRAACCTTACC; reverse: ACGGGCRGTGWGTRCAA) and V4 18S rRNA gene (18S; forward: CYGCGGTAATTCCAGCTC; reverse: AYGGTATCTRATCRTCTTYG) library preparation and sequencing. Samples were multiplexed using a dual-indexing approach and sequenced using an Illumina MiSeq with paired-end 300 + 300 bp reads. All PCR procedures and Illumina sequencing details were as previously described (Comeau et al., 2016; Yurgel et al., 2017). All sequences generated in this study are available in the NCBI sequence read archive under the accession numbers PRJNA434066 (16S) and PRJNA434067 (18S).

### Sequencing Data Processing

Sequence data processing and OTU picking were described in our earlier work (Yurgel et al., 2017). In short, the Microbiome Helper standard operating procedure was used to process and analyze the sequencing data (Comeau et al., 2017). Overlapping paired-end reads were stitched together using PEAR (v0.9.6) (Zhang et al., 2014). FASTX-Toolkit (v0.0.14) (Gordon and Hannon, 2010) was to filter out reads that had a quality score less than 30 at >10% of positions. In addition, we filtered out reads shorter than 400 bp that did not contain matching 59 and 39 sequences to the appropriate forward and reverse primers with BBMap (v35.85) (Bushnell, 2014). Chimeric reads were to screen out by running USEARCH (v6.1) (Edgar et al., 2011; Bushnell, 2014) with the options mindiv = 1.5 and minh = 0.2.

### OTU (Operational Taxonomic Unit) Picking and Statistical Analyses

Following these filtering steps, we ran open-reference OTU picking using QIIME wrapper scripts (Caporaso et al., 2010). Specifically, SortMeRNA [v2.0-dev; (Kopylova et al., 2012)] was used for the reference OTU picking steps (with sortmerna_coverage = 0.8) and sumaclust (v1.0.00) (Mercier et al., 2013) for the *de novo* OTU picking steps (with 10% of the failures sub-sampled). We filtered out OTUs that contained fewer than 0.1% of the total sequences in order to compensate for MiSeq run-to-run bleed-through (see Comeau et al., 2016). Alpha-diversity (richness and Chao1) and beta-diversity (weighted UniFrac distance) (Lozupone et al., 2011) metrics were generated using QIIME. Variations in sample groupings explained by weighted UniFrac beta-diversity distances (Adonis tests, 999 permutations) were run in QIIME to calculate how sample groupings are related to microbial community structure. Analysis of taxonomic profiles and Principal Coordinate Analysis (PCoA) were performed using the STAMP software package (Parks et al., 2014). Corrected *P*-values (*q*-values) were calculated based on Benjamini–Hochberg FDR multiple test correction.

### Co-occurrence Network Construction and Analysis

The co-occurrence analysis was performed using the CCREPE (Compositionality Corrected by REnormalization and PErmutation) R package (Schwager, in press), which has previously been used to construct co-occurrence networks from microbial sequencing data (Gevers et al., 2014; Vazquez-Baeza et al., 2016). This network uses a novel similarity measure, the N-dimensional checkerboard score (NC-score) (Stone and Roberts, 1990), which is particularly appropriate to compositions derived from microbial community sequencing data. Microbes found in less than 5% of samples were removed from the analysis. The taxa represented by less than 1% of the reads in all samples were also removed. First, the co-occurrence and co-exclusion patterns in the samples were scored. The resulted were filtered to remove non-statistically significant relationships. We generated 12 networks based on strong correlations with *p*-values < 0.01, 0.001, 0.0001 and Bonferroni cut-offs at the genus, family and order levels. The data were loaded into Cytoscape 3.4.0 (Shannon et al., 2003) and used to calculated node statistics, such as degree, betweenness centrality, and closeness centrality. We used the Excel functions NORM.DIST to fit a normal distribution to degree, betweenness centrality or closeness centrality parameters to identify the values above which nodes can be considered outliers, corresponding to *p* < 0.1. Nodes with degree, betweenness centrality and closeness centrality above *p*-value = 0.1 for all three parameters in at least two out of four correlation networks based on *p*-values < 0.01, 0.001, 0.0001 and Bonferroni cut-offs were considered to be hub taxa. The networks were visualized with Cytoscape and were represented as graphs with microbial groups as vertices/nodes and the edges as interaction types.

## Results

### Composition of Wild Blueberry Root Microbial Communities

A total of 5,569,830 high-quality 18S sequences and 595,992 high-quality 16S sequences were obtained from 49 root samples from plants from managed (MngRoot) and natural habitats (FrstRoot). The plant-derived OTUs *Archaeplastida* and *Chloroplast* were represented by 5,332,133 18S and 29,411 16S reads, respectively. For direct comparison of diversity and structure of microbial communities across environmental niches, the raw sequencing data from root samples from plans from managed (MngRoot) and natural habitats (FrstRoot) were combined with the raw sequencing data obtained from corresponding rhizosphere samples from plans from managed (MngRhizo) and natural habitats (FrstRhizo), and bulk soil samples from managed (MngBulk) and natural habitats (FrstBulk) (Yurgel et al., 2017) for processing, OTU picking and statistical analyses.

#### Eukaryotic Communities

**FIGURE 1:**
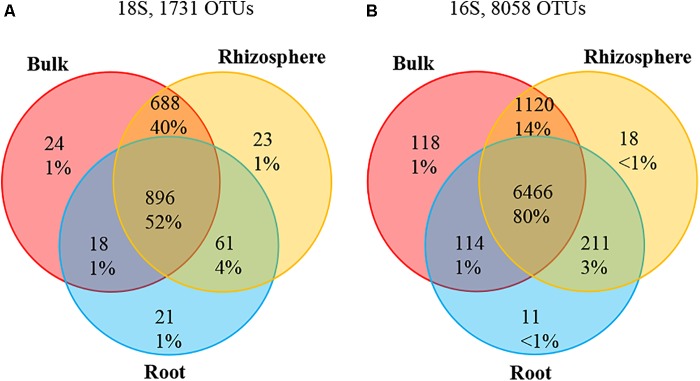
Venn diagram showing specific and shared OTUs across bulk, rhizosphere, and root niches. **(A)** 18S rRNA; **(B)** Bacteria16S rRNA.

A total of 7,036,418 high-quality 18S sequences were obtained from root, rhizosphere (MngRhizo, FrstRhizo) and bulk (MngBulk, FrstBulk) samples. To remove plant-derived OTUs, 18S sequences annotated as *Archaeplastida/Plantae* and unassigned OTUs were filtered out. A total of 1,328,201 high-quality 18S sequences remained in the dataset. After normalization to a depth of 1,922 reads (the depth of the smallest dataset after the 8 lowest samples were removed), 253,704 reads were retained, including 80,724 high-quality 18S sequences representing root community from 42 samples. These reads were distributed among 1,731 OTUs across all environmental niches and among 996 OTUs across root samples at 97% identity (**Figure 1A**). Around 52% (896) of OTUs were shared among bulk soil, rhizosphere and root habitats, while only 1% of OTUs were specific to either bulk soil, rhizosphere or root niches. A significant proportion of OTUs (688 OTUs, ∼40%) was shared among only bulk and rhizosphere.

**FIGURE 2:**
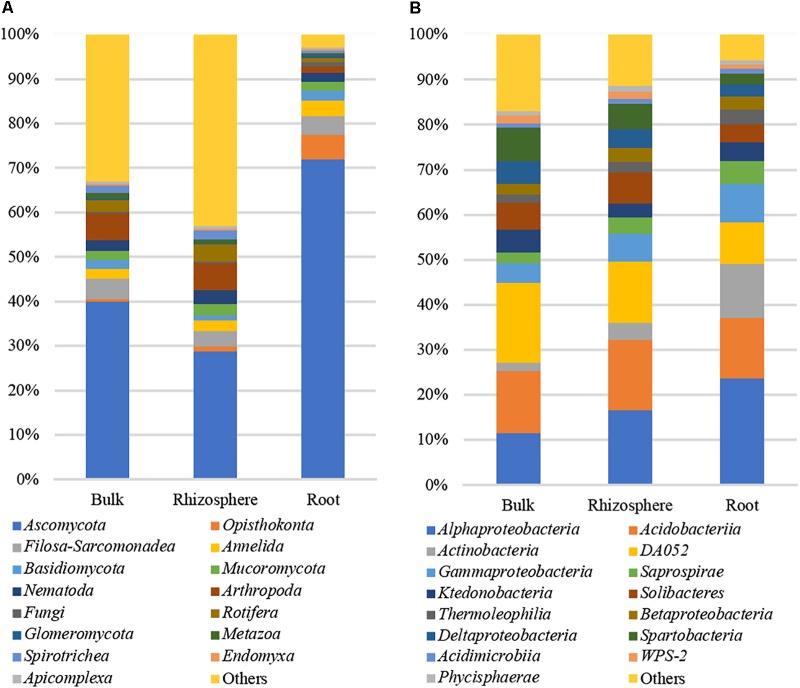
Microbial taxa identified in the study. **(A)** Bacteria16S rRNA; **(B)** 18S rRNA.

Across all environmental niches 1,201 non-fungal OTUs including *Alveolata* (343 OTUs), *Metazoa* (286 OTUs), *Cercozoa* (283 OTUs), *Hacrobia* (120 OTUs), *Stramenopiles* (68 OTUs), and *Nematoda* (64 OTUs) were identified. The top 15 most relatively abundant root-associated eukaryotic classes (**Figure 2A**) represented ∼93% of all reads identified in the root samples and only 67 and 57% of all 18S reads identified in the bulk and rhizosphere samples, respectively. Around 92% of the total read identified in root samples were classified as *Opisthokonts* division. Of this, fungi were represented by 76% of the reads, including 72% of *Ascomycota* and 2% of *Basidiomycota* and *Mucoromycota* each (**Figure 2A**). Other fungal taxa identified in the study included *Glomeromycota*, *Mucoromycotina*, and *Chytridiomycota*. Most relatively abundant non-fungal root-associated eukaryotes included *Metazoa*, ringed worms *Annelida* (4%, 7 OTUs), *Nematoda* (2%, 12 OTUs), *Arthropoda* (1.4%, 6 OTUs), and *Cercozoa* (4%, 30 OTUs).

#### Bacterial Communities

A total of 3,232,834 high-quality 16S sequences were obtained from root, rhizosphere and bulk samples. The reads annotated as *Chloroplast* were filtered, which resulting in a total of 3,186,706 high-quality 16S sequences remaining. The datasets were normalized to the depth of the smallest dataset, 7,136 reads, and 349,664 and 335,392 reads were retained in rhizosphere and bulk samples, respectively. The root community was represented by 349,664 high-quality 16S sequences across 49 samples. These reads were distributed among 8,058 OTUs across all environmental niches of which 6,802 OTUs were also found in root samples at 99% identity (**Figure 1B**). More than 80% (6,466 OTUs) were shared among three environmental habitats. Bulk, rhizosphere, and root-specific OTUs accounted for 1.5, 0.2, and 0.1% of total OTUs, respectively, and 14% of OTUs (1,120) were shared among bulk and rhizosphere. The top 15 most relatively abundant root associated bacterial classes represented ∼94% of all 16S rRNA reads identified in the root samples and ∼83 and 89% of all reads identified in the bulk and rhizosphere samples, respectively. The most abundant bacterial classes found in root samples were Proteobacteria (37%), including *Alphaproteobacteria* (23%) and *Gammaproteobacteria* (8%), *Acidobacteria* (26%), *Actinobacteria* (16%), *Bacteroidetes Saprospirae* (5%), *Chloroflexi Ktedonobacteria* (4%), and *Verrucomicrobia Spartobacteria* (2%) (**Figure 2B**).

### Transition of Microbial Communities From Rhizosphere to Root

Visualization of dissimilarity between eukaryotic communities across all environmental niches using Principal Coordinate Analysis (PCA) showed clear separation between soil bulk/rhizosphere (MngBulk, FrstBulk, MngRhizo, and FrstRhizo) and root (MngRoot and FrstRoot) communities (Supplementary Figure S1A). An analysis of the strength and statistical significance of sample groupings (Adonis test) indicated that niche (bulk, rhizosphere, and root) is associated with the structure of the eukaryotic community (*R*^2^ = 0.18, *p* < 0.001). Even stronger strength of sample groupings was detected when only rhizosphere and root niches were considered (*R*^2^ = 0.23, *p* < 0.001). Pairwise comparisons of Chao1 richness, Simpson evenness and Shannon diversity revealed a significant decrease in alpha-diversity of root eukaryotic communities compared to rhizosphere and bulk communities (Supplementary Figure S2A). Considering taxonomic groups, the relative abundance of a number of other eukaryotic taxa, such as *Arthropoda*, *Dinophyceae*, *Prymnesiophyceae*, *Spirotrichea*, and *Urochordata* (**Figure 2A** and Supplementary Figure S3A) was significantly lower in the root compared to rhizosphere. Only taxa represented by >50 and >250 18S and 16S reads, respectively, were considered. The relative abundance of fungi was decreased in rhizosphere compared to bulk soil, but increased in roots. In particular, the relative abundance of *Pezizomycotina*, which comprises most of the ascomycetous pathogens and mutualists, was significantly higher in root compared to rhizosphere. This included the classes *Sordariomycetes*, *Leotiomycetes*, containing many plant pathogens and mycorrhizal fungi, putative ericoid mycorrhizal fungi *Lachnum* (Glawe, 2008; Walker et al., 2011; Bizabani and Dames, 2015), and potential plant growth promoting dark septate endophyte *Phialocephala* (Rodriguez et al., 2009; Lukesova et al., 2015; Schlegel et al., 2016).

Similarly, to the analysis of eukaryotic comminutes, the PCoA showed clear separation between bulk, rhizosphere and root bacterial communities (Supplementary Figure S1B). The analysis of strength and statistical significance of sample groupings indicated that, niche (bulk, rhizosphere, and root) influenced the structure of the bacterial community (*R*^2^ = 0.26, *p* < 0.001). However, less strength of sample groupings was detected when only rhizosphere and root niches were considered (*R*^2^ = 0.17, *p* < 0.001). Similarly, to eukaryotic comminutes, Chao1 richness, Simpson evenness and Shannon diversity was significantly decrease in root associated bacterial communities compared to rhizosphere and bulk communities (Supplementary Figure S2B). Considering taxonomic groups, the relative abundances of several bacterial taxa, including *Chthoniobacteriales*, *Acidobacteriales*, *Ellin6513*, *Solibacterales*, and *Syntrophobacteriales* were significantly decreased in root compared to rhizosphere (Supplementary Figure S3B). The relative abundance of *Ellin6513* was also decreased in rhizosphere compared to bulk soil. Conversely, the relative abundances of *Saprospirales*, *Actinomycetales*, *Rhizobiales*, and *Xanthomonadales* had increased relative abundances with proximity to plant, with the lower relative abundances in bulk soil and the highest in root (Supplementary Figure S3B).

### Effect of Management and Field Production

Management had minor effect on root eukaryotes (Adonis test, *R*^2^ = 0.10, *p* < 0.05) and there were no differences in the relative abundances of eukaryotes detected between MngRoot and FrstRoot samples. However, when bulk, rhizosphere and root samples were analyzed together, arbuscular mycorrhizal fungi *Glomeromycota* (Davison et al., 2015) and grazers *Nematoda* were relatively more abundant and animal and fungal pathogen, *Kickxellomycota* (Spatafora et al., 2016), was relatively less abundant in forest compared to fields (Supplementary Figure S4A). Only taxa represented by >50 and >250 18S and 16S reads, respectively, were considered. Compared to eukaryotes, management has stronger effect on root bacterial communities (Adonis test, *R*^2^ = 0.14, *p* < 0.01). Bacterial orders *Chthoniobacterales*, *Ellin6513*, *Solirubrobacterales*, and *Thermogemmatisporales* had lower relative abundances, while orders *Rhodospirillales* and *Solibacterales* had higher relative abundances in FrstRoot compared to MngRoot samples (Supplementary Figure S4B). Additionally, differences in relative abundances of *Chthoniobacterales*, *Ellin6513*, *Thermogemmatisporales*, and *Rhodospirillales* were also detected between MngRhizo and FrstRhizo samples (Supplementary Figure S4B). When all niches from managed habitats were analyzed together, minor but statistically significant correlation between field fruit production and the structure of eukaryotic (Adonis test, *R*^2^ = 0.04, *p* < 0.05) but not bacterial communities (Adonis test, *R*^2^ = 0.02, *p* > 0.05) was detected. The fields with lower fruit yield had higher relative abundance of parasitic fungi *Cryptomycotina* (Letcher et al., 2017), gliding bacterivores/algaevores *Glissomonadida* (*Cercozoa*) (Howe et al., 2009) and *Vampyrellida* (*Rhizaria*) (Hess, 2017) (Supplementary Figure S5). Only taxa represented by >50 18S were considered.

### Inter-Kingdom Correlation in Wild Blueberry Microbiomes

**FIGURE 3:**
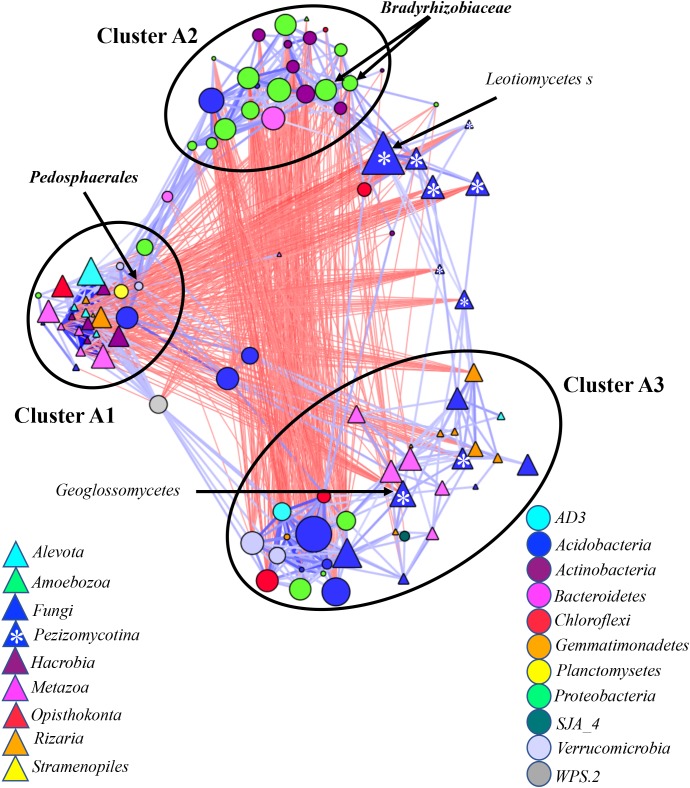
Co-occurrence network generated by measuring abundance co-correlation between microbial taxa from root and rhizosphere and bulk soil. Correlation base network analysis showing potential interactions between bacterial, fungal, and protists genera. The size of the node is proportional to a taxon’s average relative abundance across all the samples. The lines connecting nodes (edges) represent positive (blue) or negative (red) co-occurrence relationship. The intensity of the color and the length of the edges represent the strength of correlation. The taxa shown in the figure are hub taxa, which were identified as those that were significantly more central based on the measurements of degree, betweenness centrality and closeness centrality (*p* > 0.1 based on normal distribution fit). The taxa in bold were found as hub taxa at least in two co-occurrence networks base on the abundances of microorganisms grouped at the genus, family or order levels.

We generated a co-occurrence network by correlating relative abundances between microbial taxa found in ≥5% of samples from root, rhizosphere and bulks soil, with taxa grouped at the genus (**Figure 3**), family (Supplementary Figure S6A) and order levels (Supplementary Figure S6B). We used a Bonferroni cut-off to remove correlations with low *p*-values. These remaining correlations were used to construct a combined “edge-weighted spring embedded” co-occurrence network views in which positive correlations (blue) are pulling samples together forming clusters, while negative correlations (red) are pushing the samples apart.

For all three networks, three main microbial clusters were visually detected. Cluster A1 was mostly occupied by eukaryotes *Metazoa*, *Hacrobia*, *Alveolata*, and *Radiolaria* (**Figure 3** and Supplementary Figures S6A,B), many of which have previously been associated with aquatic environments. These eukaryotes negatively correlated with a taxon in *Glissomonadida* from Clusters A3 and *Pezizomycotina*, which was scattered across network based on grouping at the genus level (**Figure 3**) or stood alone in the networks based on grouping at the family and order level (Supplementary Figures S6B). Bacterial taxa found in this cluster include *Verrucomicrobia*, *Planctomycetes*, and *Candidatus Solibacter*. The eukaryotes had the higher combined relative abundance in rhizosphere compared to bulk soil and root (Supplementary Figure S7A).

Clusters A2 was mostly represented by bacterial taxa from *Alpha-, Beta-, and Gammaproteobacteria, Bacteroidetes*, and *Actinobacteria*. This cluster had a strong negative correlation with *Ascomycota* and bacterial taxa from Clusters 3 (**Figure 3** and Supplementary Figures S6A,B). Clusters A2 exhibited an increase in combined relative abundance with the shift from bulk to rhizosphere and from rhizosphere to root (Supplementary Figure S7A). Fungi, including potential pathogens, predators *Cercozoa*, *Arthropoda*, *Nematoda*, and *Rotifers*, and bacterial, *Acidobacteria, Chloroflexi*, and *Verrucomicrobia* were most abundant taxa belonging to Clusters 3 (**Figure 3** and Supplementary Figures S6A,B). The combined relative abundances of both kingdoms in this cluster were negatively affected by proximity of the plant (Supplementary Figure S7A).

**FIGURE 4:**
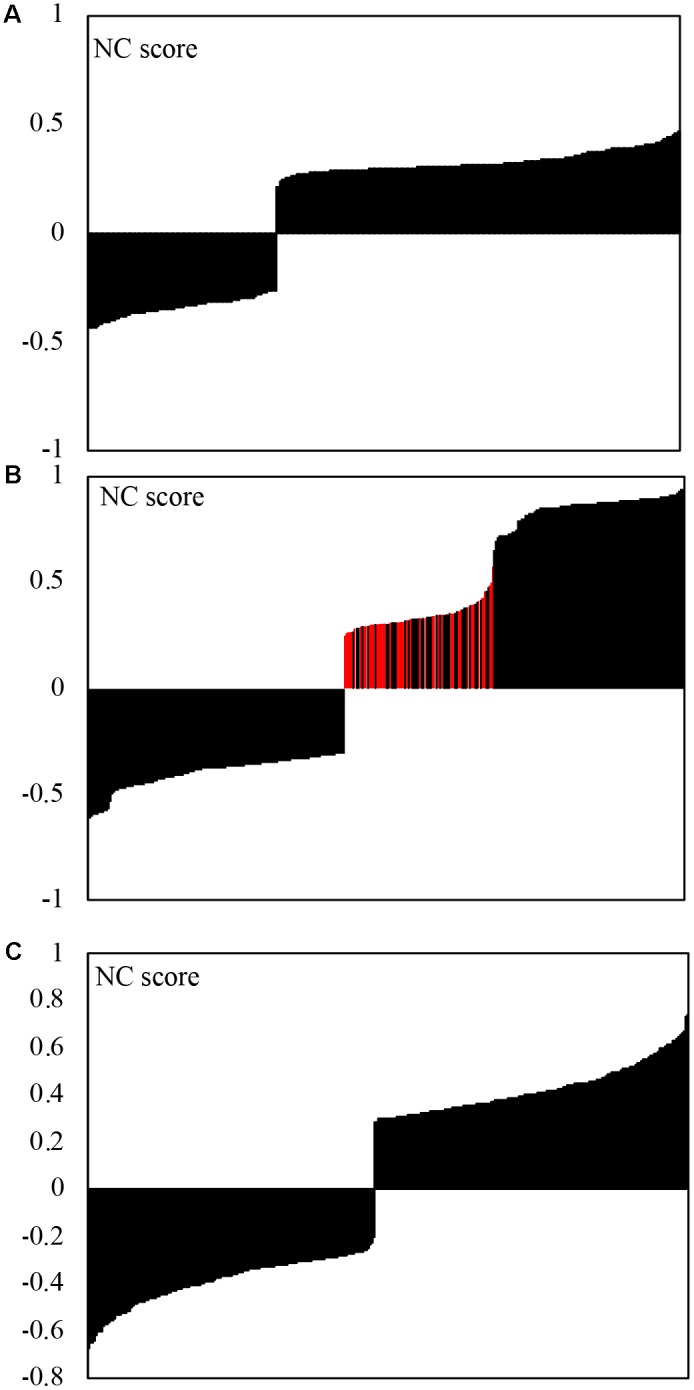
Strength of correlation (NS-score) obtained by analysis of correlations between microbial genera using the CCREPE (Compositionality Corrected by REnormalization and PErmutation) R package. *p*-value Bonferroni cut-off was used to select strong correlations. Negative NS scores represent negative correlations. **(A)** Inter-kingdom (211) correlations; **(B)** Eukaryotic (375) correlations, red bars represent positive fungal/fungal and fungal/protists correlations; **(C)** Bacterial (422) correlations.

When Bonferroni cut-off *p*-values were used to select strong correlations, inter-kingdom correlations were represented by ∼21% of all correlations, with a higher proportion of positive correlations (∼68%) compared to negative ones (32%) (**Figure 4A**). Within each kingdoms, eukaryotic and bacteria correlations were represented by 42 and 37%, respectively. Within kingdoms, the positive and negative correlations were relatively evenly distributed (**Figures 4B,C**), with 48 and 43% of negative correlations within eukaryotes and within bacteria, respectively. The positive correlations between eukaryotic organisms exhibited a clear separation into two groups with low correlation strength (NC score < 0.58) and high correlation strength score (NC score < 0.65). All correlations with fungi were located in the group with low correlation strength (red colored bars in **Figure 4B**) with average NC score significantly lower than that of non-fugal correlations (*p* < 10^-28^).

### Co-occurrence Network in Bulk and Rhizosphere Soil Communities

**FIGURE 5:**
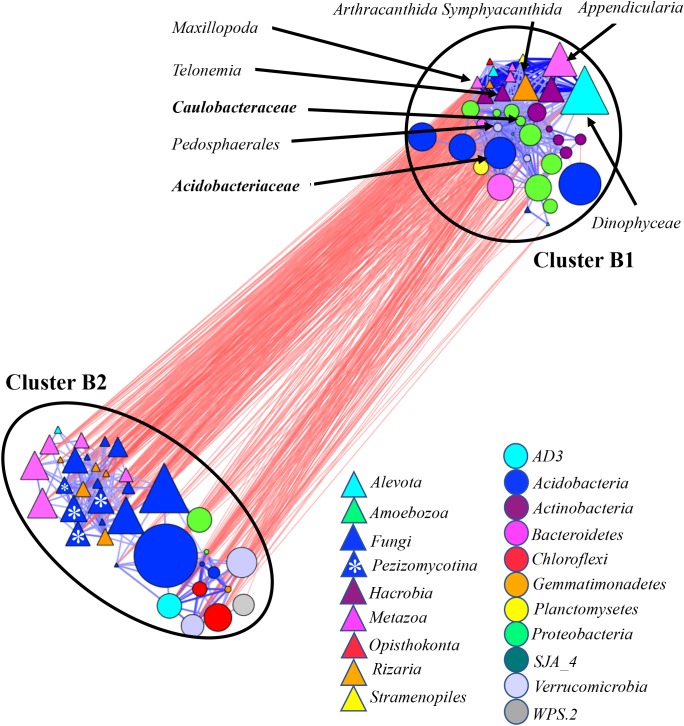
Co-occurrence network generated by measuring abundance co-correlation between microbial taxa from root and rhizosphere and bulk soil. Correlation base network analysis showing potential interactions between bacterial, fungal, and protists genera. The size of the node is proportional to a taxon’s average relative abundance across all the samples. The lines connecting nodes (edges) represent positive (blue) or negative (red) co-occurrence relationship. The intensity of the color and the length of the edges represent the strength of correlation. The taxa shown in the figure are hub taxa, which were identified as those that were significantly more central based on the measurements of degree, betweenness centrality and closeness centrality (*p* > 0.1 based on normal distribution fit). The taxa in bold were found as hub taxa at least in two co-occurrence networks base on the abundances of microorganisms grouped at the genus, family or order levels.

**FIGURE 6:**
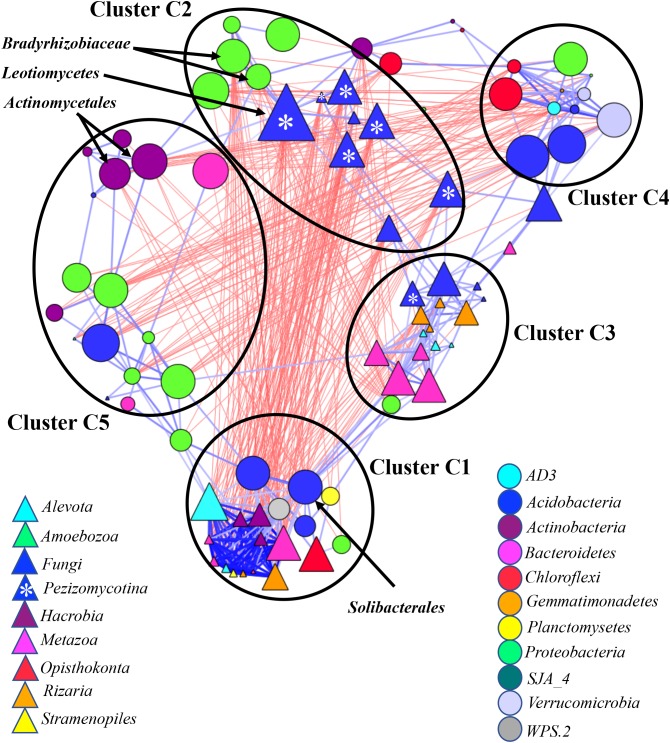
Co-occurrence network generated by measuring abundance co-correlation between microbial taxa from root and rhizosphere. Correlation base network analysis showing potential interactions between bacterial, fungal, and protists genera. The size of the node is proportional to a taxon’s average relative abundance across all the samples. The lines connecting nodes (edges) represent positive (blue) or negative (red) co-occurrence relationship. The intensity of the color and the length of the edges represent the strength of correlation. The taxa shown in the figure are hub taxa, which were identified as those that were significantly more central based on the measurements of degree, betweenness centrality and closeness centrality (*p* > 0.1 based on normal distribution fit). The taxa in bold were found as hub taxa at least in two co-occurrence networks base on the abundances of microorganisms grouped at the genus, family or order levels.

Similarly, we generated co-occurrence sub-networks by considering bacterial and eukaryotic communities in (i) bulk and rhizosphere soil (soil-associated) (**Figure 5**) and (ii) rhizosphere and root (root-associated) microbiomes (**Figure 6**). Two microbial clusters were detected in the network considering the soil-associated microbiota (**Figure 3**). Cluster B1 was occupied by eukaryotes *Metazoa*, *Hacrobia*, *Alveolata*, and *Radiolaria* and bacteria *Acidobacteria*, *Actinobacteria*, *Bacteroidetes*, and *Proteobacteria*. These taxa were more abundant in rhizosphere compared to bulk soil (Supplementary Figure S7B). Eighty percent of eukaryotic and 86% of bacterial taxa, found in this cluster, were also found in the Clusters A1 and A2 in the network based on microbial communities across all environment niches (**Figure 3**). On average, the taxa found in Cluster B2 in the network considering the soil-associated microbiome were more abundant in bulk soil compared to rhizosphere and many of the taxa, found in this cluster (**Figure 5**) were also found in Cluster A3, considering microbial communities across all environment niches (**Figure 3**). Fungi were also a part of Cluster B2 in the network considering bulk and rhizosphere soil.

### Co-occurrence Network in Rhizosphere and Root Communities

The network considering abundance co-correlation in the root-associated microbiome exhibited striking complexity (**Figure 6**). One strongly interconnected cluster (Cluster C1) and C4 loosely interconnected clusters were detected in the network. The Cluster C1 was mostly occupied by eukaryotes, some of which had been primarily associated with aquatic environments. Aquatic bacteria *Planctomycetes* and *Acidobacteria* were also found in the cluster. The Cluster C1 had negative correlation with *Bradyrhizobiaceae* and *Pezizomycotina* - the major taxa comprising Cluster C2. Both kingdoms from Cluster C1 were more abundant in rhizosphere compared to root (Supplementary Figure S7C) and reverse tendency was detected in Cluster C2. Cluster C3 included predators *Filosa Sarcomonadea*, *Metazoa* (*Arthropoda*, *Nematoda*, and *Rotifera*) and *Alveolata* and several potential parasitic fungi. Clusters C4 and C5 were mostly represented by bacteria.

### Identification of Hub Taxa in Microbial Networks

For identification of hub microbes, we generated co-occurrence networks based on strong correlations with *p*-values < 0.01, 0.001, 0.0001 and Bonferroni cut-offs for microbial taxa grouped at genus, family or order levels. 12 networks were generated for each of 3 types of communities: (i) across all environmental niches, (ii) associated with bulk and rhizosphere soil, and (iii) associated with rhizosphere and plant root. The multiple *p*-values were used since it has been previously shown that the strength of cut-offs affects network structure (Kurtz et al., 2015). Similarly, in our analysis we found differences in the parameters of the nodes from the networks based on different *p*-value cut-offs. For each network, we identified nodes with significantly higher degree, betweenness centrality or closeness centrality based on normal distribution fit with *p* < 0.1 for all three parameters using at least two out of four correlation cut-offs. These nodes were considered as potential outliers.

Several potential outliers were identified in the networks considering microbial communities across all environmental niches and based on different grouping of the taxa (Supplementary Table S2). The bacterial genera *Bradyrhizobiaceae* and *Pedosphaerales* and the fungi *Pezizomycotina*, *Geoglossomycetes*, and *Leotiomycetes* were highly connected (**Figure 3**). Combined together, these four microbial taxa were directly connected to 30 eukaryotic and 34 bacterial taxa, which represented 67% of all nodes in network based on correlations with *p*-values with Bonferroni cut-offs. *Bradyrhizobium* and *Pedosphaerales* were supported at all taxonomic levels (**Figure 3** and Supplementary Figures S6A,B) and were directly connected to 21 (43%) eukaryotic and 29 (62%) bacterial taxa grouped at genera level, based on correlations with *p*-values with Bonferroni cut-offs. In the co-occurrence network considering the soil-associated microbiome and based on the microbial taxa grouped at genera level, eukaryotic taxa *Dinophyceae*, *Telonemia*, *Maxillopoda*, and *Appendicularia* and bacterial taxa *Acidobacteriaceae*, *Caulobacteraceae*, and *Pedosphaerales* were highly connected. *Acidobacteriaceae*, *Caulobacteraceae* were also found as hub taxa at least in one other co-occurrence networks based on different grouping (Supplementary Table S3 and **Figure 5**). Considering the root-associated microbiome, *Bradyrhizobiaceae*, *Actinomycetales*, *Solibacterales*, *Pedosphaerales*, and the fungi *Pezizomycotina Leotiomycetes* were identified as hub taxa in at least two co-occurrence networks (Supplementary Table S4 and **Figure 6**).

## Discussion

The concept of holobiont encompasses a complex network of functions and symbiotic interactions between host–plant and its microbiome. Over the past several years an understanding the bacterial and fungal communities as components of the holobiont and their role in plant nutrient acquisition and resistance to biotic and abiotic stresses has progressed significantly (Vandenkoornhuyse et al., 2015). However, a full comprehension of plant holobiont concept is missing an explanation of how the non-fungal eukaryotic microorganisms fit in this complex network of plant–microbiome interactions. To elucidate the role of soil eukaryotes and prokaryotes in plant-associated microbiome we investigated wild blueberry root microbiomes containing 996 eukaryotic and 6,802 bacterial OTUs, respectively. This community was mostly comprised by endophytes with possible minor proportion of root-epiphytic microorganisms. The microbiome was combined with previously identified bulk soil and rhizosphere microbiomes (Yurgel et al., 2017) resulting in a total of 530 fungal, 1201 non-fungal eukaryotic, and 8058 bacterial OTUs across all environmental niches. This comprehensive dataset enabled us to evaluate the role of non-fungal eukaryotes in shaping the interactions between blueberry plants and soil microbes.

### Eukaryotic Community

Similar to previous reports (Leach et al., 2017), fungi were the most abundant eukaryotic taxa in the wild blueberry root microbiome. These fungi included a number of potential mycorrhizal fungal taxa, such as *Pezizomycotina, Glomeromycota*, *Mucoromycotina*, and *Chytridiomycota* (Kohout et al., 2012; Davison et al., 2015; van der Heijden et al., 2015; Orchard et al., 2017), putative ericoid mycorrhizal fungi *Lachnum* (Walker et al., 2011; Bizabani and Dames, 2015; Zhang et al., 2016), and potential plant growth promoting endophyte *Phialocephala* (Rodriguez et al., 2009; Lukesova et al., 2015; Schlegel et al., 2016). We were able to identify 30, 12, 7, and 6 individual OTUs belonging to *Cercozoa*, *Nematoda*, *Annelida*, and *Arthropoda*, respectively. In total these microorganisms were represented by ∼12% of the high quality 18S reads and were the most abundant non-fungal eukaryotes in the root microbiome.

### Bacterial Community

It has been previously shown that bacteria belonging to the three main phyla, *Proteobacteria, Actinobacteria*, and *Bacteroidetes*, are the most abundant taxa in plant rhizosphere and endosphere, while the phylum *Acidobacteria* is often excluded from these compartments (Hacquard et al., 2015; Leach et al., 2017). Similarly, *Alphaproteobacteria* and *Gammaproteobacteria, Actinobacteria*, and *Bacteroidetes* were among the five most abundant bacterial taxa in wild blueberry roots and were enriched along the soil-endosphere continuum. Although *Acidobacteria* was the second most abundant phylum in root microbiome, its abundance was significantly decreased in roots compared to rhizosphere. The exclusion of *Acidobacteria* from endophytic compartment has been reported in other plant microbiomes (Bulgarelli et al., 2013).

### Transition of Microbiome Along the Soil-Endosphere Continuum

Bulk soil microbiome serves as a foundation for plant-associated microbiome (Shakya et al., 2013; Schreiter et al., 2014). In agreement with this notion, more than 52% of eukaryotic and 80% of bacterial OTUs identified in this study are found in all three environmental niches while only 1% of OTUs were unique to root associated microbiome. Rhizosphere is the first habitat with a strong plant influence on microbial communities. However, the transition from root surface to interior provides the strongest species sorting effect, demanding microbial specialization necessary for invasion and survival inside plant tissue and, as a result, a significant decrease in community diversity (Hacquard et al., 2015; Vandenkoornhuyse et al., 2015). Our data also indicated a significant decrease in bacterial and eukaryotic diversity in root microbiome compared to rhizosphere and bulk soil, probably as a result of this specialization process.

As previously reported in the wild blueberry rhizosphere samples, plant-influenced species sorting has a stronger effect on bacteria than on eukaryotes (Yurgel et al., 2017). Here, we detected the opposite species sorting effect in the root microbiome. A lower proportion of eukaryotic OTUs than bacterial OTUs were shared between all three environments. Moreover, the 15 most abundant eukaryotes in the root were at significantly lower relative abundance in the other two environments, which was not the case for the most abundant bacteria in the roots. In agreement with this finding, niche (rhizosphere vs. root) correlated with 23% of eukaryotic and 17% of bacterial community variation. The specific effect of species sorting in root microbiome due to host resulted in an increase in relative abundance of fungi adapted to plant-associated life-style, such as *Lachnum*, *Phialocephala*, and *Sordariomycete*s, as well as lichen-forming fungi *Lecanorales*, *Cetradonia linearis* and *Cladonia*. In contrast, the relative abundances of microscopic animals, protists and algae were decreased along the soil-endosphere continuum in the root, probably because of strong impact of host defense system on community establishment and low adaptation of non-fungal eukaryotes to host-plant defense responses.

### The Link Between Field Fruit Production and Community Structure

Since we did not have detailed information about fruit yield in the managed fields used as sampling sites, we assigned each field with high or low fruit yield parameter, based on the assessment of field production by blueberry growers. Our data showed minor but statistically significant correlation between field fruit production and the structure of eukaryotic community. More specifically, we found that parasitic fungi *Cryptomycotina* (Letcher et al., 2017), gliding bacterivores/algaevores *Glissomonadida* (*Cercozoa*) (Howe et al., 2009) and *Vampyrellida* (*Rhizaria*) (Hess, 2017) were more abundant in the fields with low fruit yield across all three environmental niches. The presence of these microorganisms might directly or indirectly affect plant health through the disease development or/and depletion of microbial taxa with beneficial effect on plant development and production. However, more detailed studies are required to verify this hypothesis, which include disease survey of the fields and increase of the number of sampling sites for better resolution of differences in the structure of microbiomes.

Previously we showed that the aggregate difference in forest *vs.* managed systems influenced bacterial communities in blueberry rhizospheres, but had less effect on eukaryotic communities (Yurgel et al., 2017). A similar tendency of stronger effect of management on bacterial communities compared to eukaryotic communities was detected in blueberry roots. The main factors involved in the differentiation of microbial community between managed and natural habitats might include previous pruning of the plants, use of soil-applied fertilizers and pest management practices, which resulted in higher growth rates and fruit production of managed stands compared to forest stands (Yurgel et al., 2017).

### Community Correlation Networks

To further evaluate the rules guiding the assembly of plant-associated microbiota, we generated bacterial and eukaryotic community correlation networks, which incorporated different levels of host–plant influence. In this model the combined bulk and rhizosphere communities (soil-associated microbiome) were considered to be under lower plant influence compared to combined rhizosphere and root communities (root-associated microbiome). The combination of microbial communities across all environmental niches in a single correlation networks provided a complementary view of microbe–microbe and plant–microbe interaction in a broader ecological setting. Previous studies showed that the bacterial network is more complex in root-associated microbiome than in bulk soil (Cordero and Datta, 2016; Shi et al., 2016). We also found that microbial network, comprising both bacterial and eukaryotic microorganisms became more complex along the soil-endosphere continuum. Only two visually well-defined clusters emerged in the soil-associated co-occurrence network, while four loosely and one tightly associated cluster emerged in the root-associated co-occurrence network.

The existence of extensive mutualistic interactions among plant-associated microorganisms is a well-known concept (Hallam and McCutcheon, 2015). Previously within kingdoms of plant-associated microorganisms, more positive than negative correlations were detected, while more negative than positive correlation were detected between microbes from different kingdoms (Agler et al., 2016). Our data showed a more even distribution of positive and negative correlations within kingdoms suggesting that co-exclusion or competition also plays an important role in shaping plant associated microbiomes. A previous study of inter-kingdom correlations showed that soil protists formed distinct clusters that link a range of bacterial and fungal taxa (Xiong et al., 2017). In all networks constructed in this study, tightly associated clusters comprising both bacterial and eukaryotes organisms were identified. Moreover, a higher proportion of positive correlations compared to negative ones was detected between microbes from different kingdoms, which emphasizes the importance of mutualistic interactions in inter-kingdom networks.

### Identification of Hub Taxa in the Microbial Networks

Across all environmental niches bacteria, *Bradyrhizobium* and *Pedosphaerales*, and fungi, *Pezizomycotina Geoglossomycetes*, and *Leotiomycetes*, were identified as hub taxa, suggesting an important role of these microorganisms in wild blueberry soil ecosystem.

#### Hub Taxa in Plant-Associated Microbiome

*Bradyrhizobium, Pedosphaerales*, and *Leotiomycetes*, were also hubs in the network considering root-associates microbiome. The ability of some highly interconnected microbial taxa to link host factors to plant microbiome variation has been recently tested (Agler et al., 2016). *Bradyrhizobium* genus is one of the most ubiquitous microorganisms in soils from across the world and plays a critical role in soil fertility. These bacteria have been used as a model organism to study the impact of environmental factors on soil microbiota (Shah and Subramaniam, 2017). The organisms from the *Bradyrhizobium* genus are able to promote plant growth by producing the plant growth promoting compounds indole-3-acetic acid and 1-amino-cyclopropane-1-carboxylic acid deaminase as well as fixating nitrogen inside plant tissues (Piromyou et al., 2017). The genus *Leotiomycetes* contains many plant pathogens and mycorrhizal fungi (Glawe, 2008; Walker et al., 2011; Bizabani and Dames, 2015) and therefore might have a profound effect on soil and plant health and, as a result also an effect on the microbiomes associated with these niches. *Actinobacteria Actinomycetales*, and *Acidobacteria Solibacterales* were also identified as hubs in root-associated microbiome. *Actinobacteria* is one of the most abundant taxa in plant rhizosphere and endosphere (Hacquard et al., 2015; Leach et al., 2017) and play a major role soil fertility and plant health. *Actinomycetes* are capable to improve the availability of nutrients and minerals in soils, suppress some pathogens and promote plant health (Bhatti et al., 2017).

#### Hub Taxa in Soil-Associated Microbiome

In the network considering abundance co-correlation in soil-associated microbiome, non-fungal eukaryotic taxa *Dinophyceae*, *Telonemia*, *Maxillopoda*, and *Appendicularia* and bacterial taxa *Acidobacteriaceae*, *Caulobacteraceae*, and *Pedosphaerales* were identified as hubs. *Acidobacteria*, is one of the most abundant bacterial taxa in soils, especially in acidic conditions predicted to be involved in soil nutrient cycling (Kielak et al., 2016). Ecological interactions *Acidobacteria* with *Proteobacteria* (Kielak et al., 2016) and with soils protists (Xiong et al., 2017) had been reported. While recently it was shown that *Dinophyceae* is an important member of soil protistan communities (Bates et al., 2013; Yurgel et al., 2017), little is known about the role of *Telonemia*, *Maxillopoda*, and *Appendicularia* in soil microbiome. Therefore, our findings emphasize the complexity of soil ecology and the need for more research aimed to understand the inter-kingdom interactions in soil and plant-associated communities.

## Conclusion

Our analyses of eukaryotic and bacterial communities associated with wild blueberry ecosystems allowed us to better understand the plant-influenced species sorting effect on soil bacteria and fungi and gain insights into the effect of this process on non-fungal eukaryotes. This study also extends our knowledge about the effect of aggregate difference in forest vs. managed systems on microbial communities and identified several eukaryotic taxa that are potentially linked to the decrease in fruit production in wild blueberry agricultural ecosystems. Our data indicated that bacterial and eukaryotic interactions become more complex along the soil-endosphere continuum and included extensive mutualistic inter-kingdom. We also identified several potential hub taxa with important roles in soil fertility and/or plant–microbe interaction and indicated the potential role of these taxa in the interconnection between soils and plant health and overall microbial community structure.

## Author Contributions

SY and ML obtained funding. SY and DP designed the study and collected the samples. SY and AD processed the samples and performed the data pre-possessing. SY performed the bioinformatics analyses and wrote the manuscript. GD assisted with the bioinformatics analyses. SY, ML, and DP analyzed and discussed the results. SY, GD, ML, and DP participated in the production and the final version of the manuscript.

## Conflict of Interest Statement

The authors declare that the research was conducted in the absence of any commercial or financial relationships that could be construed as a potential conflict of interest.

## Footnotes

1 http://cgeb-imr.ca/

## References

[B1] Agler M. T. Ruhe J. Kroll S. Morhenn C. Kim S. T. Weigel D. (2016). Microbial hub Taxa link host and abiotic factors to plant microbiome variation. *PLoS Biol.* 14:e1002352. 10.1371/journal.pbio.1002352 26788878 PMC4720289

[B2] Bates S. T. Clemente J. C. Flores G. E. Walters W. A. Parfrey L. W. Knight R. (2013). Global biogeography of highly diverse protistan communities in soil. *ISME J.* 7 652–659. 10.1038/ismej.2012.147 23235291 PMC3578557

[B3] Bell D. J. Rowland L. J. Zhang D. Drummonda F. A. (2009). Spatial genetic structure of lowbush blueberry, *Vaccinium angustifolium*, in four fields in Maine. *Botany* 87 932–946. 10.1094/PHYTO-02-16-0093-R 27775501

[B4] Bhatti A. A. Haq S. Bhat R. A. (2017). Actinomycetes benefaction role in soil and plant health. *Microb. Pathog.* 111 458–467. 10.1016/j.micpath.2017.09.036 28923606

[B5] Bizabani C. Dames J. (2015). Effects of inoculating *Lachnum* and *Cadophora* isolates on the growth of *Vaccinium corymbosum*. *Microbiol. Res.* 181 68–74. 10.1016/j.micres.2015.08.005 26640054

[B6] Bulgarelli D. Schlaeppi K. Spaepen S. Ver Loren van Themaat E. Schulze-Lefert P. (2013). Structure and functions of the bacterial microbiota of plants. *Annu. Rev. Plant Biol.* 64 807–838. 10.1146/annurev-arplant-050312-120106 23373698

[B7] Busby P. E. Soman C. Wagner M. R. Friesen M. L. Kremer J. Bennett A. (2017). Research priorities for harnessing plant microbiomes in sustainable agriculture. *PLoS Biol.* 15:e2001793. 10.1371/journal.pbio.2001793 28350798 PMC5370116

[B8] Bushnell B. (2014). *BBMap: A Fast, Accurate, Splice-Aware Aligner.* Technical report LBNL-7065E Berkeley, CA: Ernest Orlando Lawrence Berkeley Natl Lab.

[B9] Caporaso J. G. Kuczynski J. Stombaugh J. Bittinger K. Bushman F. D. Costello E. K. (2010). QIIME allows analysis of high-throughput community sequencing data. *Nat. Methods* 7 335–336. 20383131 10.1038/nmeth.f.303 PMC3156573

[B10] Comeau A. M. Douglas G. M. Langille M. G. (2017). Microbiome helper: a custom and streamlined workflow for microbiome research. *mSystems* 2:e00127-16. 10.1128/mSystems.00127-16 28066818 PMC5209531

[B11] Comeau A. M. Vincent W. F. Bernier L. Lovejoy C. (2016). Novel chytrid lineages dominate fungal sequences in diverse marine and freshwater habitats. *Sci. Rep.* 6:30120. 10.1038/srep30120 27444055 PMC4957111

[B12] Cordero O. X. Datta M. S. (2016). Microbial interactions and community assembly at microscales. *Curr. Opin. Microbiol.* 31 227–234. 10.1016/j.mib.2016.03.015 27232202 PMC5157693

[B13] Davison J. Moora M. Opik M. Adholeya A. Ainsaar L. Ba A. (2015). FUNGAL SYMBIONTS. Global assessment of arbuscular mycorrhizal fungus diversity reveals very low endemism. *Science* 349 970–973. 10.1126/science.aab1161 26315436

[B14] Drummond F. Smagula J. Annis S. Yarborough D. (2009). Organic Wild Blueberry Production. *MAFES Bull.* 852:43.

[B15] Eaton L. J. (1988). *Nutrient Cycling in Lowbush Blueberries.* Halifax: Dalhousie University.

[B16] Edgar R. C. Haas B. J. Clemente J. C. Quince C. Knight R. (2011). UCHIME improves sensitivity and speed of chimera detection. *Bioinformatics* 27 2194–2200. 10.1093/bioinformatics/btr381 21700674 PMC3150044

[B17] Fierer N. (2017). Embracing the unknown: disentangling the complexities of the soil microbiome. *Nat. Rev. Microbiol.* 15 579–590. 10.1038/nrmicro.2017.87 28824177

[B18] Geisen S. (2016). The bacterial-fungal energy channel concept challenged by enormous functional versatility of soil protists. *Soil Biol. Biochem.* 102 22–25.

[B19] Geisen S. Rosengarten J. Koller R. Mulder C. Urich T. Bonkowski M. (2015a). Pack hunting by a common soil amoeba on nematodes. *Environ. Microbiol.* 17 4538–4546. 10.1111/1462-2920.12949 26079718

[B20] Geisen S. Tveit A. T. Clark I. M. Richter A. Svenning M. M. Bonkowski M. (2015b). Metatranscriptomic census of active protists in soils. *ISME J.* 9 2178–2190. 10.1038/ismej.2015.30 25822483 PMC4579471

[B21] Gevers D. Kugathasan S. Denson L. A. Vazquez-Baeza Y. Van Treuren W. Ren B. (2014). The treatment-naive microbiome in new-onset Crohn’s disease. *Cell Host Microbe* 15 382–392. 10.1016/j.chom.2014.02.005 24629344 PMC4059512

[B22] Glawe D. A. (2008). The powdery mildews: a review of the world’s most familiar (yet poorly known) plant pathogens. *Annu. Rev. Phytopathol.* 46 27–51. 10.1146/annurev.phyto.46.081407.104740 18680422

[B23] Gordon A. Hannon G. (2010). *FASTX-Toolkit: FASTQ/A Short-Reads Preprocessing Tools.* Available at: http://hannonlab.cshl.edu/fastx_toolkit

[B24] Grossmann L. Jensen M. Heider D. Jost S. Glucksman E. Hartikainen H. (2016). Protistan community analysis: key findings of a large-scale molecular sampling. *ISME J.* 10 2269–2279. 10.1038/ismej.2016.10 26859769 PMC4989302

[B25] Hacquard S. Garrido-Oter R. Gonzalez A. Spaepen S. Ackermann G. Lebeis S. (2015). Microbiota and host nutrition across plant and animal kingdoms. *Cell Host Microbe* 17 603–616. 10.1016/j.chom.2015.04.009 25974302

[B26] Hall I. V. Aalders L. E. Nickerson N. L. Vander Kloet S. P. (1979). The biological flora of Canada. I. *Vaccinium angustifolium* Ait., sweet lowbush blueberry. *Canad. Field Naturalist* 93 415–430.

[B27] Hallam S. J. McCutcheon J. P. (2015). Microbes don’t play solitaire: how cooperation trumps isolation in the microbial world. *Environ. Microbiol. Rep.* 7 26–28. 25721597 10.1111/1758-2229.12248

[B28] Hess S. (2017). Hunting for agile prey: trophic specialisation in leptophryid amoebae (Vampyrellida, Rhizaria) revealed by two novel predators of planktonic algae. *FEMS Microbiol. Ecol.* 93:fix104. 10.1093/femsec/fix104 28922804

[B29] Howe A. T. Bass D. Vickerman K. Chao E. E. Cavalier-Smith T. (2009). Phylogeny, taxonomy, and astounding genetic diversity of Glissomonadida ord. nov., the dominant gliding zooflagellates in soil (Protozoa: Cercozoa). *Protist* 160 159–189. 10.1016/j.protis.2008.11.007 19324594

[B30] Kielak A. M. Barreto C. C. Kowalchuk G. A. van Veen J. A. Kuramae E. E. (2016). The ecology of *Acidobacteria*: moving beyond genes and genomes. *Front. Microbiol.* 7:744 10.3389/fmicb.2016.00744 PMC4885859 27303369

[B31] Kohout P. Sykorova Z. Ctvrtlikova M. Rydlova J. Suda J. Vohnik M. (2012). Surprising spectra of root-associated fungi in submerged aquatic plants. *FEMS Microbiol. Ecol.* 80 216–235. 10.1111/j.1574-6941.2011.01291.x 22224638

[B32] Kopylova E. Noe L. Touzet H. (2012). SortMeRNA: fast and accurate filtering of ribosomal RNAs in metatranscriptomic data. *Bioinformatics* 28 3211–3217. 10.1093/bioinformatics/bts611 23071270

[B33] Kurtz Z. D. Muller C. L. Miraldi E. R. Littman D. R. Blaser M. J. Bonneau R. A. (2015). Sparse and compositionally robust inference of microbial ecological networks. *PLoS Comput. Biol.* 11:e1004226. 10.1371/journal.pcbi.1004226 25950956 PMC4423992

[B34] Lareen A. Burton F. Schafer P. (2016). Plant root-microbe communication in shaping root microbiomes. *Plant Mol. Biol.* 90 575–587. 10.1007/s11103-015-0417-8 26729479 PMC4819777

[B35] Leach J. E. Triplett L. R. Argueso C. T. Trivedi P. (2017). Communication in the Phytobiome. *Cell* 169 587–596. 10.1016/j.cell.2017.04.025 28475891

[B36] Letcher P. M. Longcore J. E. James T. Y. Leite D. S. Simmons D. R. Powell M. J. (2017). Morphology, ultrastructure, and molecular phylogeny of *Rozella multimorpha*, a new species in Cryptomycota. *J. Eukaryot. Microbiol.* 65 180–190. 10.1111/jeu.12452 28749611

[B37] Lozupone C. Lladser M. E. Knights D. Stombaugh J. Knight R. (2011). UniFrac: an effective distance metric for microbial community comparison. *ISME J.* 5 169–172. 20827291 10.1038/ismej.2010.133 PMC3105689

[B38] Lukesova T. Kohout P. Vetrovsky T. Vohnik M. (2015). The potential of dark septate endophytes to form root symbioses with ectomycorrhizal and ericoid mycorrhizal middle European forest plants. *PLoS One* 10:e0124752. 10.1371/journal.pone.0124752 25905493 PMC4408093

[B39] Mercier C. Boyer F. Bonin A. Coissac E. (2013). *SUMATRA and SUMACLUST: Fast and Exact Comparison and Clustering of Sequences.* Available at: http://metabarcoding.org/sumatra

[B40] Orchard S. Hilton S. Bending G. D. Dickie I. A. Standish R. J. Gleeson D. B. (2017). Fine endophytes (*Glomus tenue*) are related to Mucoromycotina, not Glomeromycota. *New Phytol.* 213 481–486. 27768808 10.1111/nph.14268

[B41] Parks D. H. Tyson G. W. Hugenholtz P. Beiko R. G. (2014). STAMP: statistical analysis of taxonomic and functional profiles. *Bioinformatics* 30 3123–3124. 10.1093/bioinformatics/btu494 25061070 PMC4609014

[B42] Piromyou P. Greetatorn T. Teamtisong K. Tittabutr P. Boonkerd N. Teaumroong N. (2017). Potential of rice stubble as a reservoir of bradyrhizobial inoculum in rice-legume crop rotation. *Appl. Environ. Microbiol.* 83:e01488-17. 10.1128/AEM.01488-17 28916558 PMC5666126

[B43] Rodriguez R. J. White J. F. Jr. Arnold A. E. Redman R. S. (2009). Fungal endophytes: diversity and functional roles. *New Phytol.* 182 314–330. 10.1111/j.1469-8137.2009.02773.x 19236579

[B44] Schlegel M. Munsterkotter M. Guldener U. Bruggmann R. Duo A. Hainaut M. (2016). Globally distributed root endophyte *Phialocephala subalpina* links pathogenic and saprophytic lifestyles. *BMC Genomics* 17:1015. 10.1186/S12864-016-3369-8 27938347 PMC5148876

[B45] Schreiter S. Ding G. C. Heuer H. Neumann G. Sandmann M. Grosch R. (2014). Effect of the soil type on the microbiome in the rhizosphere of field-grown lettuce. *Front. Microbiol.* 5:144 10.3389/fmicb.2014.00144 PMC3986527 24782839

[B46] Schwager E. (in press). Detecting statistically significant associtations between sparse and high dimensional compositional data.

[B47] Seppey C. V. W. Singer D. Dumack K. Fournier B. Belbahri L. Mitchell E. A. D. (2017). Distribution patterns of soil microbial eukaryotes suggests widespread algivory by phagotrophic protists as an alternative pathway for nutrient cycling. *Soil Biol. Biochem.* 112 68–76.

[B48] Shade A. (2017). Diversity is the question, not the answer. *ISME J.* 11 1–6. 10.1038/ismej.2016.118 27636395 PMC5421358

[B49] Shah V. Subramaniam S. (2017). *Bradyrhizobium japonicum* USDA110: a representative model organism for studying the impact of pollutants on soil microbiota. *Sci. Total Environ.* 624 963–967. 10.1016/j.scitotenv.2017.12.185 29275259

[B50] Shakya M. Gottel N. Castro H. Yang Z. K. Gunter L. Labbe J. (2013). A multifactor analysis of fungal and bacterial community structure in the root microbiome of mature *Populus deltoides* trees. *PLoS One* 8:e76382. 10.1371/journal.pone.0076382 24146861 PMC3797799

[B51] Shannon P. Markiel A. Ozier O. Baliga N. S. Wang J. T. Ramage D. (2003). Cytoscape: a software environment for integrated models of biomolecular interaction networks. *Genome Res.* 13 2498–2504. 14597658 10.1101/gr.1239303 PMC403769

[B52] Shi S. Nuccio E. E. Shi Z. J. He Z. Zhou J. Firestone M. K. (2016). The interconnected rhizosphere: high network complexity dominates rhizosphere assemblages. *Ecol. Lett.* 19 926–936. 10.1111/ele.12630 27264635

[B53] Spatafora J. W. Chang Y. Benny G. L. Lazarus K. Smith M. E. Berbee M. L. (2016). A phylum-level phylogenetic classification of zygomycete fungi based on genome-scale data. *Mycologia* 108 1028–1046. 27738200 10.3852/16-042 PMC6078412

[B54] Stone L. Roberts A. (1990). The checkerboard score and species distributions. *Oecologia* 85 74–79. 10.1007/BF00317345 28310957

[B55] van der Heijden M. G. Martin F. M. Selosse M. A. Sanders I. R. (2015). Mycorrhizal ecology and evolution: the past, the present, and the future. *New Phytol.* 205 1406–1423. 10.1111/nph.13288 25639293

[B56] van Overbeek L. S. Saikkonen K. (2016). Impact of bacterial-fungal interactions on the colonization of the endosphere. *Trends Plant Sci.* 21 230–242. 10.1016/j.tplants.2016.01.003 26821607

[B57] Vandenkoornhuyse P. Quaiser A. Duhamel M. Le Van A. Dufresne A. (2015). The importance of the microbiome of the plant holobiont. *New Phytol.* 206 1196–1206. 10.1111/nph.13312 25655016

[B58] Vazquez-Baeza Y. Hyde E. R. Suchodolski J. S. Knight R. (2016). Dog and human inflammatory bowel disease rely on overlapping yet distinct dysbiosis networks. *Nat. Microbiol.* 1:16177. 10.1038/nmicrobiol.2016.177 27694806

[B59] Walker J. F. Aldrich-Wolfe L. Riffel A. Barbare H. Simpson N. B. Trowbridge J. (2011). Diverse Helotiales associated with the roots of three species of Arctic Ericaceae provide no evidence for host specificity. *New Phytol.* 191 515–527. 10.1111/j.1469-8137.2011.03703.x 21463329

[B60] Xiong W. Jousset A. Guo S. Karlsson I. Zhao Q. Wu H. (2017). Soil protist communities form a dynamic hub in the soil microbiome. *ISME J.* 12 634–638. 10.1038/ismej.2017.171 29028001 PMC5776453

[B61] Yang H. Li J. Xiao Y. Gu Y. Liu H. Liang Y. (2017). An integrated insight into the relationship between soil microbial community and tobacco bacterial wilt disease. *Front. Microbiol.* 8:2179. 10.3389/fmicb.2017.02179 29163453 PMC5681905

[B62] Yurgel S. N. Douglas G. M. Comeau A. M. Mammoliti M. Dusault A. Percival D. (2017). Variation in bacterial and eukaryotic communities associated with natural and managed wild blueberry habitats. *Phytobiomes* 1 102–113.

[B63] Zhang J. Kobert K. Flouri T. Stamatakis A. (2014). PEAR: a fast and accurate Illumina Paired-End reAd mergeR. *Bioinformatics* 30 614–620. 10.1093/bioinformatics/btt593 24142950 PMC3933873

[B64] Zhang R. Vivanco J. M. Shen Q. (2017). The unseen rhizosphere root-soil-microbe interactions for crop production. *Curr. Opin. Microbiol.* 37 8–14. 10.1016/j.mib.2017.03.008 28433932

[B65] Zhang Y. Ni J. Tang F. Pei K. Luo Y. Jiang L. (2016). Root-associated fungi of *Vaccinium carlesii* in subtropical forests of China: intra- and inter-annual variability and impacts of human disturbances. *Sci. Rep.* 6:22399. 10.1038/srep22399 26928608 PMC4772160

[B66] Zilber-Rosenberg I. Rosenberg E. (2008). Role of microorganisms in the evolution of animals and plants: the hologenome theory of evolution. *FEMS Microbiol. Rev.* 32 723–735. 10.1111/j.1574-6976.2008.00123.x 18549407

