# Supplementary material for: Dissecting Community Structure in Wild Blueberry Root and Soil Microbiome
Source: Front Microbiol. 2018 Jun 6;9:1187. doi: 10.3389/fmicb.2018.01187 (PMC5996171; doi:10.3389/fmicb.2018.01187)
Supplement: Supplementary file 1 [file Table_1.DOCX]

Table S1. Description of samples

| Sample type, Key, Source | Number of samples | | | | | | |
| --- | --- | --- | --- | --- | --- | --- | --- |
|  | Total | Farmington | Collinwood | Debert-1 | Debert-2 | NSBI | Mt. Thom |
| Forest habitats | | | | | | | |
| Roots from the plants after collection of FrstRhizo soil, **FrstRoot**, this work | 15 | N/C | 5 | N/C | N/C | 10 | N/C |
| Bulk soil within close proximity to roots of blueberry plants from forest, **FrstBulk,** Yurgel *et al*., 2017 | 17 | N/C | 5 | N/C | N/C | 12 | N/C |
| Rhizosphere soil from roots of blueberry plants from forest, **FrstRhizo,** Yurgel *et al*., 2017 | 15 | N/C | 5 | N/C | N/C | 10 | N/C |
| Managed habitats | | | | | | | |
| Roots from the plants after collection of MngRhizo soil, **MngRoot**, this work | 34 | 5  High** | 5  Low | 10  Low | 5  High | 5  Low | 4  High |
| Bulk soil within close proximity to roots of blueberry plants from managed field, **MngBulk,** Yurgel *et al*., 2017 | 34 | 5  High | 5  Low | 10  Low | 5  High | 5  Low | 4  High |
| Rhizosphere soil from roots of blueberry plants from managed fields, **MngRhizo,** Yurgel *et al*., 2017 | 34 | 5  High | 5  Low | 10  Low | 5  High | 5  Low | 4  High |

*Not collected

**Fruit yield
