# Supplementary material for: Dissecting Community Structure in Wild Blueberry Root and Soil Microbiome
Source: Front Microbiol. 2018 Jun 6;9:1187. doi: 10.3389/fmicb.2018.01187 (PMC5996171; doi:10.3389/fmicb.2018.01187)
Supplement: Supplementary file 3 [file Table_3.DOCX]

Table S3. Hub nodes in correlational network across bulk and rhizosphere communities.

| Microbial taxa | 0.01* | 0.001 | 0.0001 | Bonferroni |
| --- | --- | --- | --- | --- |
| 366 bacteria and 219 eukaryotes taxa grouped at the genus level | | | | |
| *Dinophyceae* |  |  | X** | X |
| *Telonemia* | X |  |  | X |
| *Maxillopoda* | X | X |  |  |
| *Appendicularia* | X |  |  | X |
| *Arthracanthida.Symphyacanthida* | X |  |  | X |
| *Acidobacteriaceae* |  |  | X | X |
| *Caulobacteraceae* |  |  | X | X |
| *Pedosphaerales* |  |  | X | X |
| 251 bacteria and 134 eukaryotes taxa grouped at the family level | | | | |
| *Caulobacteraceae* |  |  | X | X |
| *Acidobacteriaceae* |  |  | X | X |
| 159 bacteria and 76 eukaryotes taxa grouped at the order level | | | | |
| *Caulobacterales* |  |  | X | X |
| *Ascomycota* |  |  | X | X |

The nodes with significantly (p<0.1 based on fitting a normal distribution) higher degree, betweenness centrality and closeness centrality in the correlation networks based on strong correlations with p-values > 0.01, 0.001, 0.0001 or Bonferroni cutoff

* - p value cutoff used to remove week correlations.

** - Nodes with the all parameters above p = 0.1
