# Supplementary material for: Dissecting Community Structure in Wild Blueberry Root and Soil Microbiome
Source: Front Microbiol. 2018 Jun 6;9:1187. doi: 10.3389/fmicb.2018.01187 (PMC5996171; doi:10.3389/fmicb.2018.01187)
Supplement: Supplementary file 5 [file Image_1.PDF]

(A)

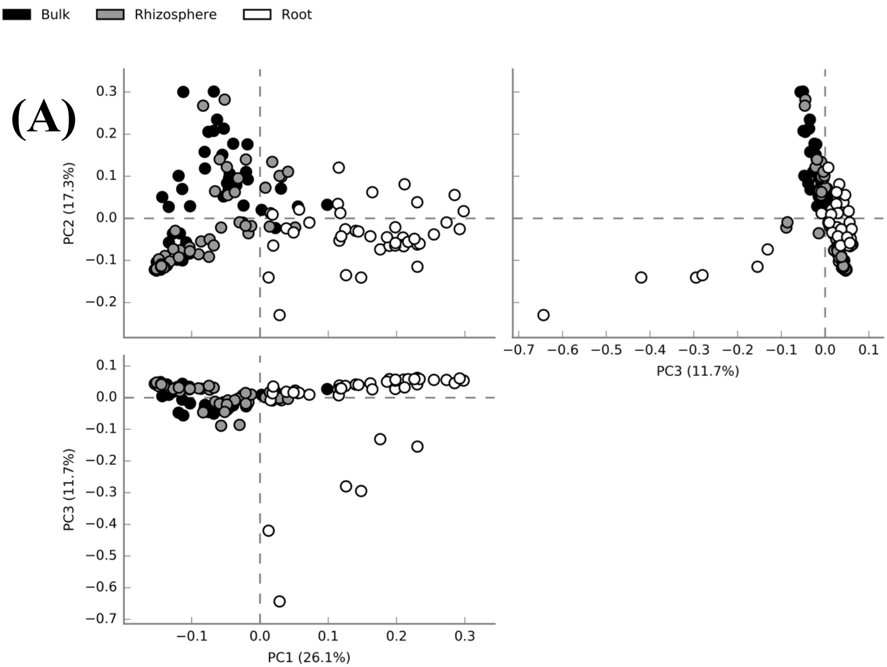

(B)

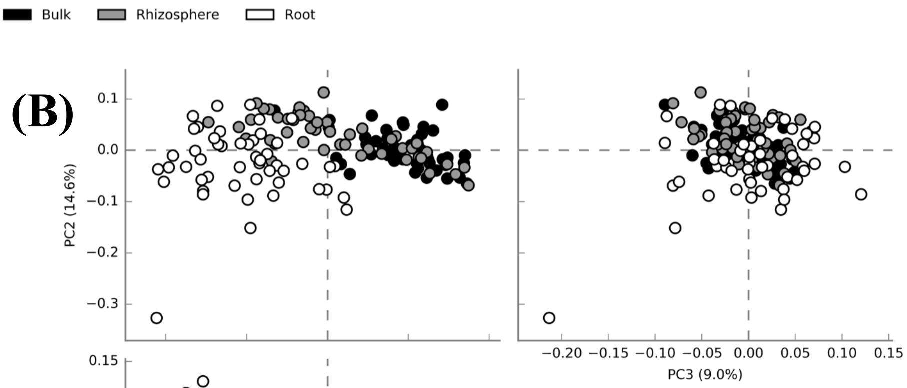

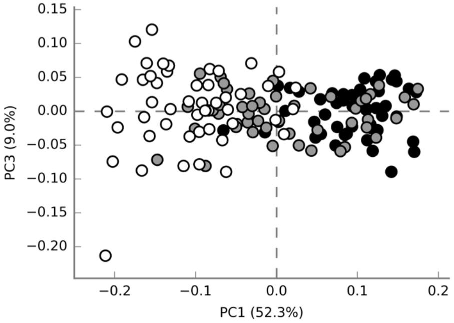

Figure S1. Principal Coordinate Analysis (PCA) of microbial communities based on (A) 18S (eukaryotic) and (B) 16S (bacterial) community Bray-Curtis dissimilarity matrix. Each point is a different sample and the colors indicate the different sampling niches.
