# Supplementary material for: Dissecting Community Structure in Wild Blueberry Root and Soil Microbiome
Source: Front Microbiol. 2018 Jun 6;9:1187. doi: 10.3389/fmicb.2018.01187 (PMC5996171; doi:10.3389/fmicb.2018.01187)
Supplement: Supplementary file 6 [file Image_2.PDF]

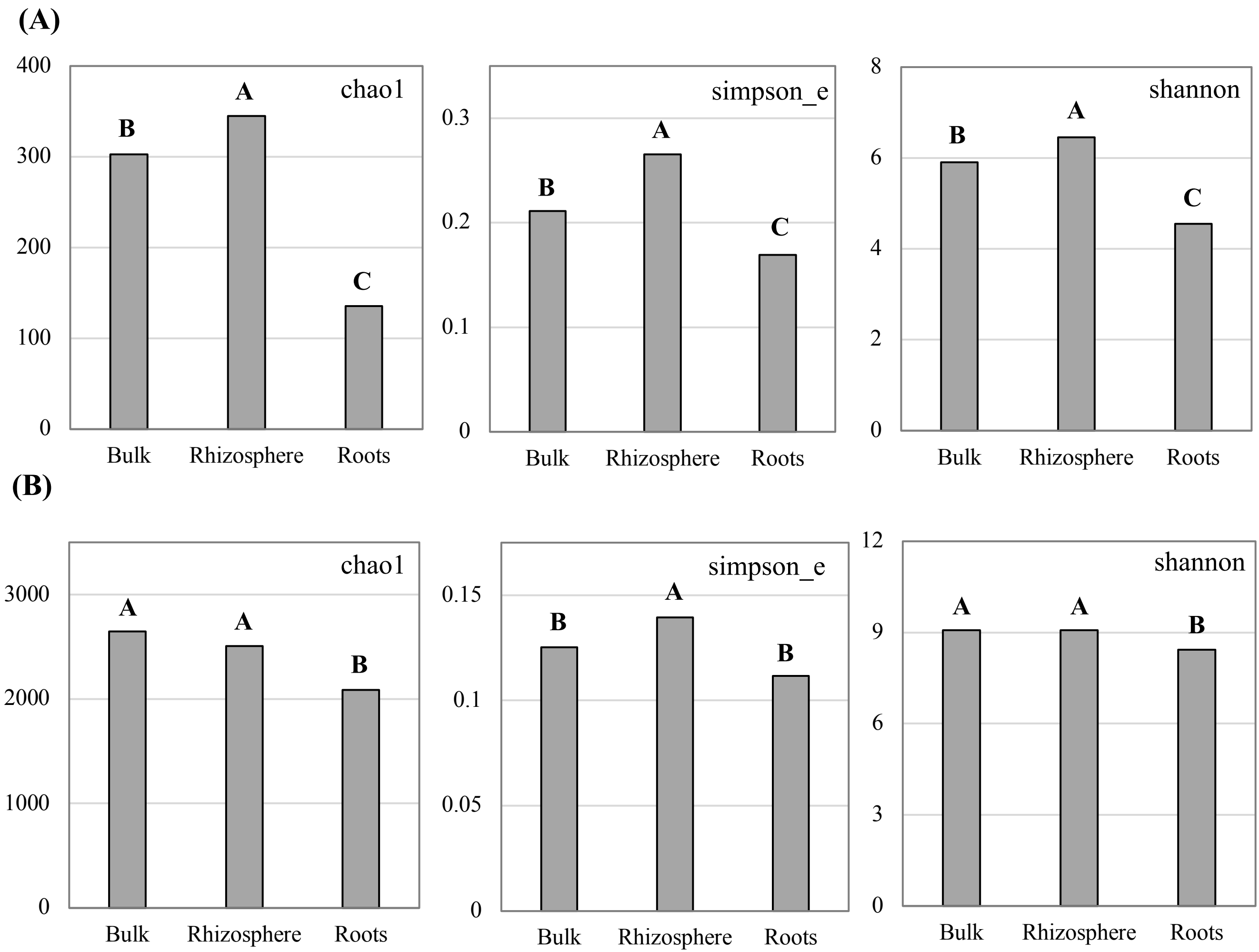

Figure S2. Estimated total species richness (chao1), Simpson Evenness (simpson_e) and Shannon Diversity (shannon). For each variable, data followed by different letters are significantly different according to Tukey's pairwise test ($p<0.05$). (A) – 18S rRNA; (B) – 16S rRNA
