# Supplementary material for: Dissecting Community Structure in Wild Blueberry Root and Soil Microbiome
Source: Front Microbiol. 2018 Jun 6;9:1187. doi: 10.3389/fmicb.2018.01187 (PMC5996171; doi:10.3389/fmicb.2018.01187)
Supplement: Supplementary file 7 [file Image_3.PDF]

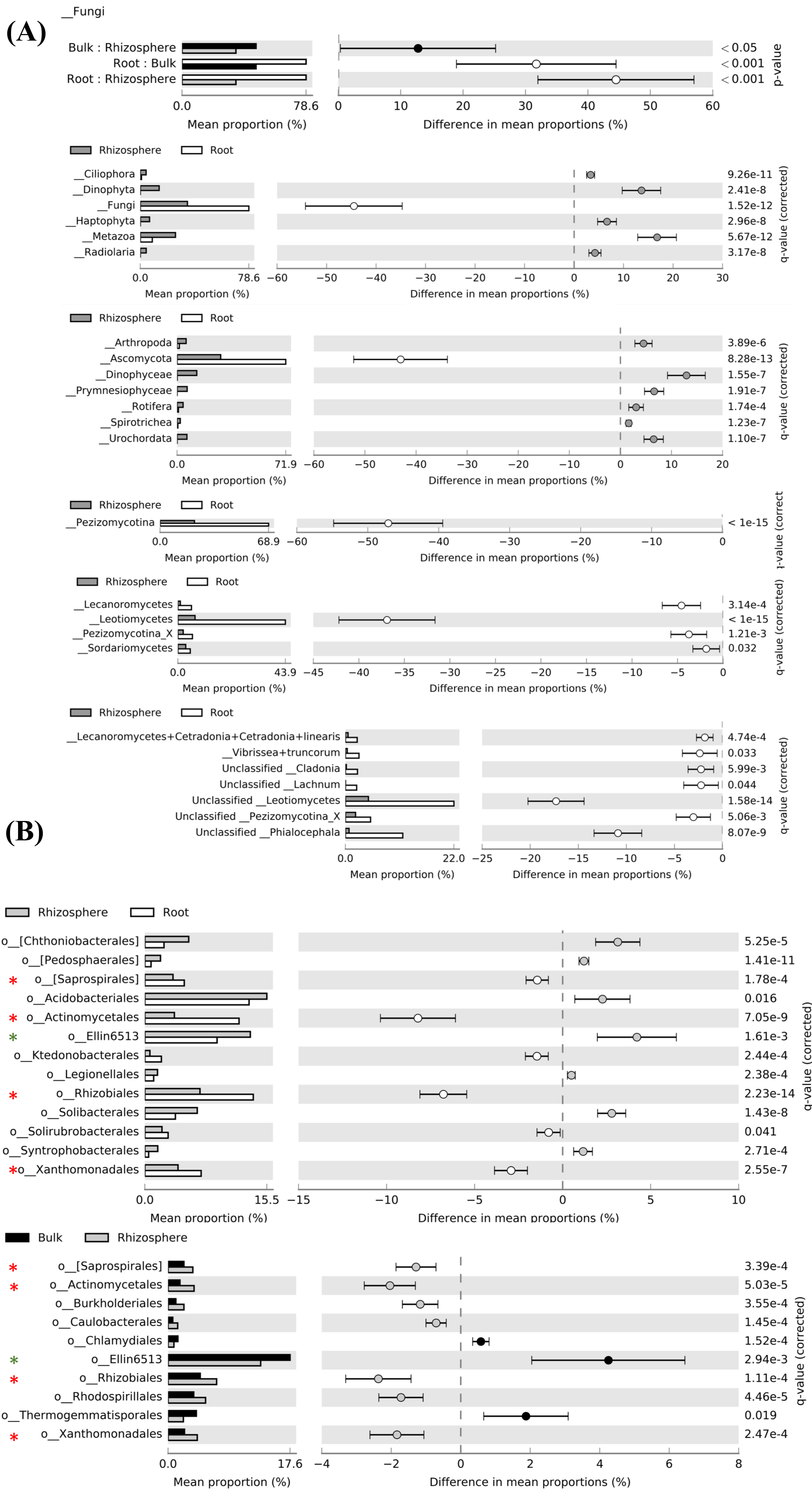

Figure S3. Microbial taxa that were significantly overrepresented in comparison between bulk, root and rhizosphere communities: (A) – Eukaryotic taxa; (B) – Bacterial taxa.
Corrected P-values (q-values) were calculated based on Benjamini-Hochberg FDR multiple test correction. Features with (Welch's t-test) q value <0.01 were considered significant and were thus retained. The stars indicate bacterial taxa with increased (red) or decreased (green) relative abundances in both root vs. rhizosphere and rhizosphere vs. bulk communities.
