# Supplementary material for: Dissecting Community Structure in Wild Blueberry Root and Soil Microbiome
Source: Front Microbiol. 2018 Jun 6;9:1187. doi: 10.3389/fmicb.2018.01187 (PMC5996171; doi:10.3389/fmicb.2018.01187)
Supplement: Supplementary file 8 [file Image_4.PDF]

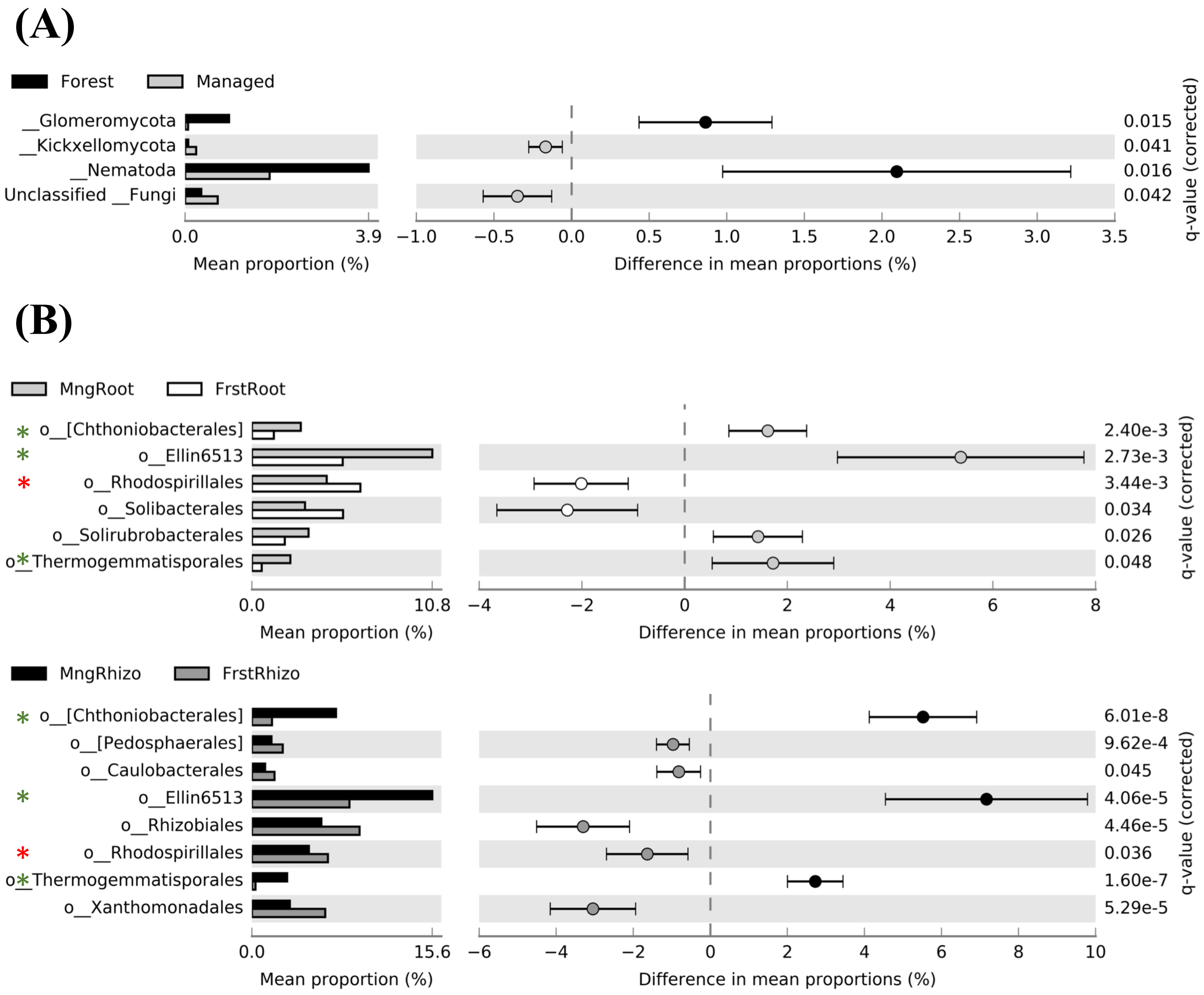

Figure S4. Microbial taxa that were significantly overrepresented in comparison between communities from managed and natural (Forest) habitats. (A) – Eukaryotic taxa; (B) – Bacterial taxa. Corrected P-values (*q*-values) were calculated based on Benjamini-Hochberg FDR multiple test correction. Features with (Welch's *t*-test) *q* value <0.01 were considered significant and were thus retained. The stars indicate bacterial taxa with increased (green) or decreased (red) relative abundances in both root and rhizosphere communities form managed habitats compared to that from natural habitats.
