# Supplementary material for: Dissecting Community Structure in Wild Blueberry Root and Soil Microbiome
Source: Front Microbiol. 2018 Jun 6;9:1187. doi: 10.3389/fmicb.2018.01187 (PMC5996171; doi:10.3389/fmicb.2018.01187)
Supplement: Supplementary file 9 [file Image_5.PDF]

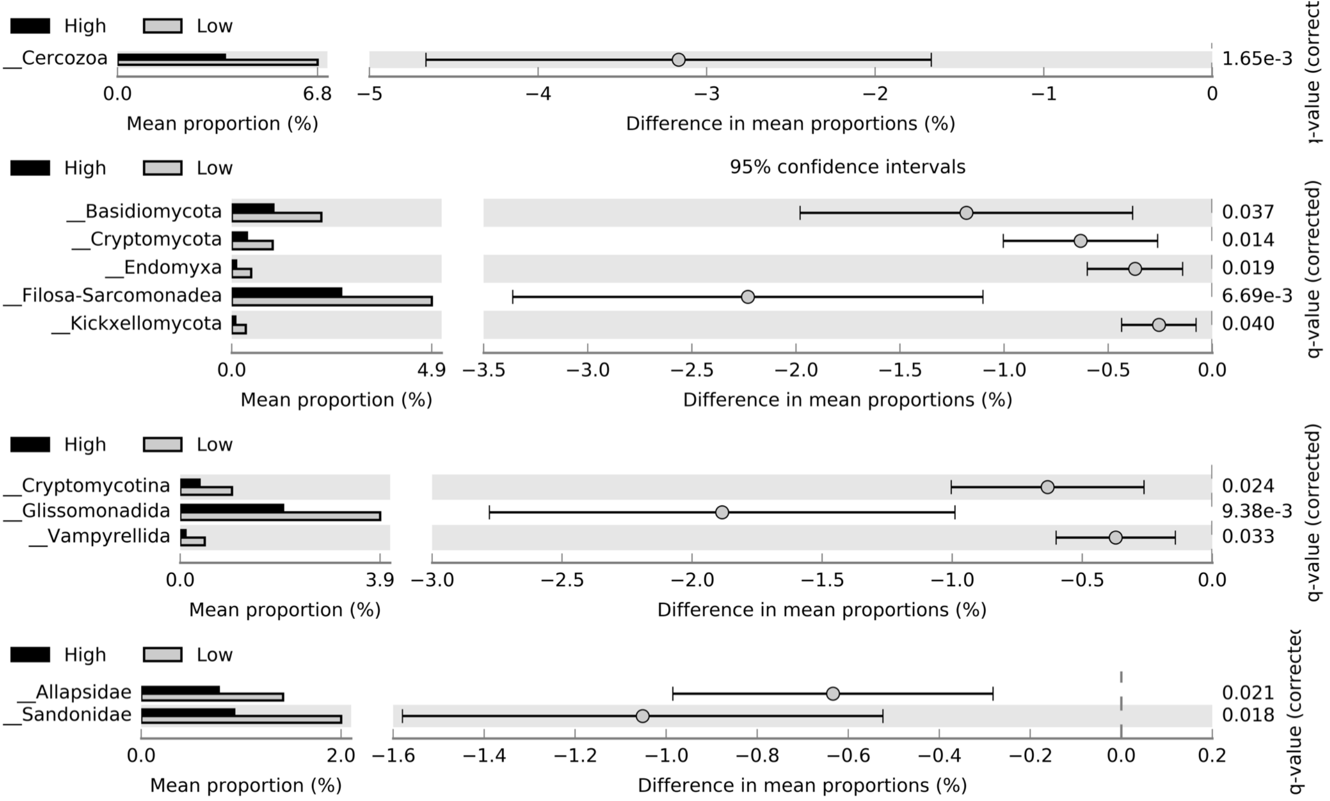

Figure S5. Microbial taxa that were significantly overrepresented in comparison between communities from high and low yield fields. Corrected P-values (*q*-values) were calculated based on Benjamini-Hochberg FDR multiple test correction. Features with (Welch's *t*-test) *q* value <0.01 were considered significant and were thus retained.
