# Supplementary material for: Dissecting Community Structure in Wild Blueberry Root and Soil Microbiome
Source: Front Microbiol. 2018 Jun 6;9:1187. doi: 10.3389/fmicb.2018.01187 (PMC5996171; doi:10.3389/fmicb.2018.01187)
Supplement: Supplementary file 10 [file Image_6.PDF]

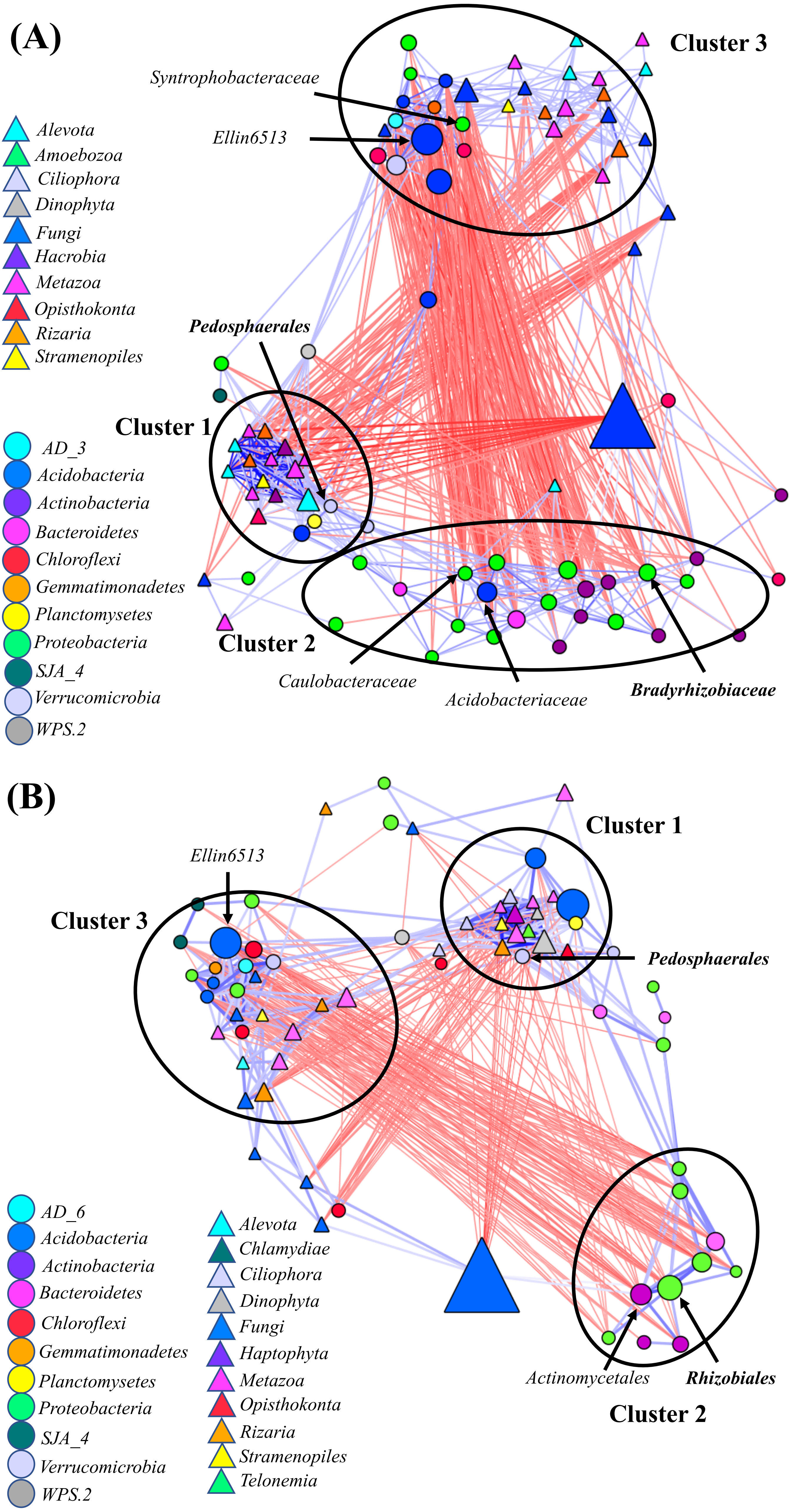

Figure S6. Correlation base network analysis of microbial communities across all environmental niches showing potential interactions between bacterial, fungal and protists grouped at (A) – family level; (B) – order level.

For network shown, p-value Bonferroni cutoff was used to select strong correlations. The size of the node is proportional to a taxon's average relative abundance across all the samples. The lines connecting nodes (edges) represent positive (blue) or negative (red) co-occurrence relationship. The intensity of the color represent the strength of correlation. The taxa shown in the figure are central taxa identified by analysis of the networks with p-value 0.01, 0.001, 0.0001 and Bonferroni cutoffs. Hub taxa were identified as those that were significantly more central based on the measurements of degree, betweeness centrality and closeness centrality ($p > 0.1$ based on normal distribution fit). The taxa in bold were found as central taxa in two co-occurrence networks base on the abundances of microorganisms grouped at the genus, family or order levels.
