# Supplementary material for: Dissecting Community Structure in Wild Blueberry Root and Soil Microbiome
Source: Front Microbiol. 2018 Jun 6;9:1187. doi: 10.3389/fmicb.2018.01187 (PMC5996171; doi:10.3389/fmicb.2018.01187)
Supplement: Supplementary file 11 [file Image_7.PDF]

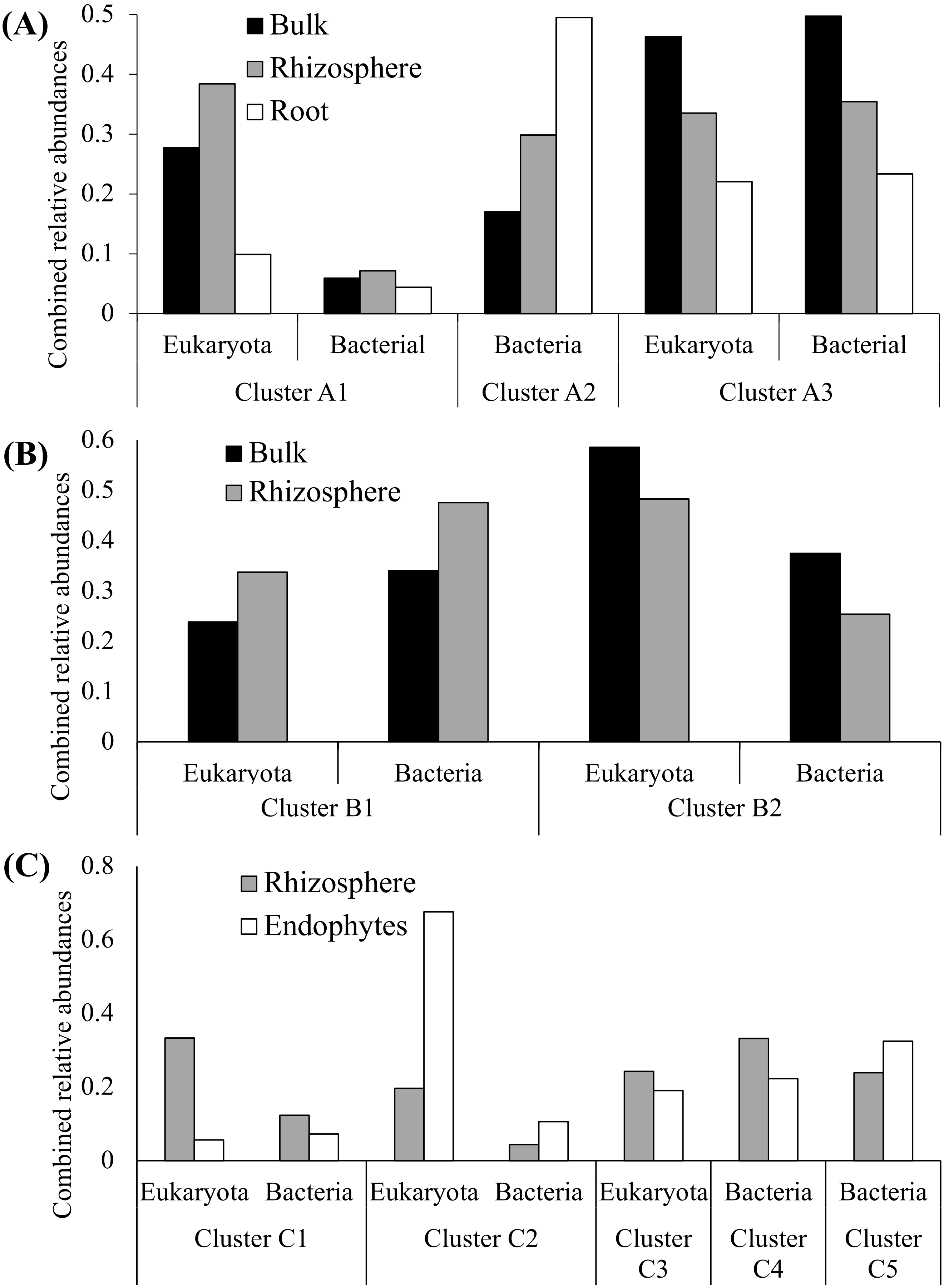

Figure S7. Combined relative abundances of microbial taxa from clusters detected in the co-occurrence networks generated by measuring abundance co-correlation between microbes grouped at the genus level. Bonferroni cut-off was used to removed correlations with low p-values. (A) – Microbial communities across all environmental niches; (B) – Microbial communities from bulk and rhizosphere; (C) – Microbial communities from rhizosphere and plant roots
